# Conformational Stability Effect of Polymeric Iron Chelators

**DOI:** 10.1016/j.isci.2019.10.022

**Published:** 2019-10-14

**Authors:** Jian Qian, Cory Berkland

**Affiliations:** 1 Department of Pharmaceutical Chemistry, The University of Kansas, Lawrence, KS 66047, USA; 2 Department of Chemical and Petroleum Engineering, The University of Kansas, Lawrence, KS 66045, United States

**Keywords:** Polymer Chemistry, Chemical Synthesis, Polymers

## Abstract

The design and synthesis of metal chelators with extraordinary metal affinities is a basic and challenging scientific problem of both fundamental and practical importance. Here, we demonstrate a “conformational stability effect” that can significantly enhance the metal affinity of ligands after conjugation to polymer chains with the ability to spontaneously adopt a specific conformation as an optimal “soft” scaffold to ensure maximum thermodynamic stability of the metal complexes. Using iron chelators as models, we show that simple conjugation of small molecule catechol ligands to a polyallylamine chain resulted in more than 8–9 orders of magnitude enhancement of the iron-binding affinity, which is comparable to that of enterobactin, the strongest iron chelator ever known. This study demonstrates that flexible polymer chelators may realize the highest possible metal affinities of the conjugated ligands owing to their ability to achieve an optimal conformation, which could advance the identification of strong metal chelators.

## Highlights

• Flexible polymer chelator adopts an optimal conformation to coordinate ferric iron
• This conformation as a soft scaffold produces lowest strain and highest iron affinity
• Polymer chelator with that soft scaffold exhibits >10^8^ enhancement on iron affinity

## Graphical Abstract

**Figure:**
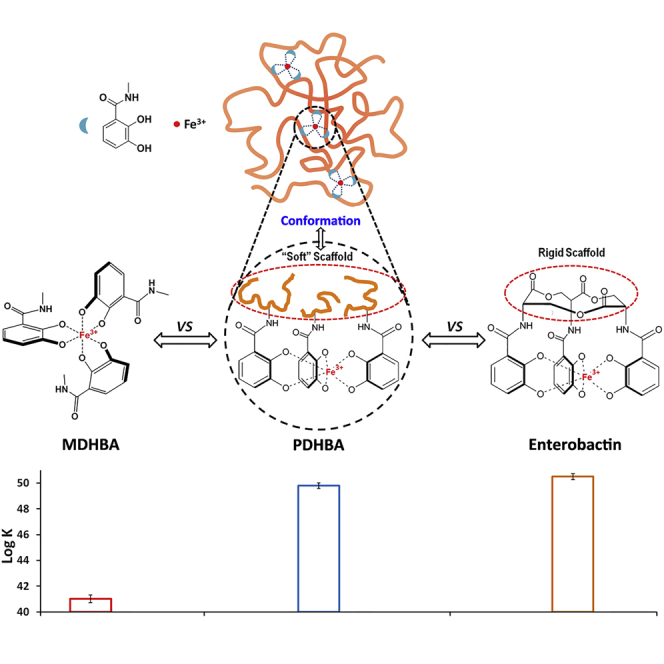

## Introduction

Metal chelators are widely used and indispensable in a variety of fields in both the laboratory and industry. Improving the metal-binding affinities of chelators is always a central problem that directly impacts the efficacies of related applications. Several principles in coordination chemistry can provide general guidance for designing metal chelators (Hancock and Martell, 1989, Haas and Franz, 2009), yet no simple and straightforward strategy is available to achieve strong metal-binding affinities. Generally, conventional metal chelators are designed by organizing functional ligands with certain linkage units (scaffolds) to be constrained in the conformation for matching the specific metal coordination geometry. Owing to the rigid characteristics of these scaffolds, the metal-ligand coordination may induce distortions of the bond angles and generate strain in the formed complexes, which can greatly impair the thermodynamic stability (Cram and Cram, 1978, Rodgers and Armentrout, 2003, Buist et al., 2010, Alvarez, 2015). An optimal scaffold should ensure that the preorganized ligands coordinate the target metal with a best fit coordination geometry that produces minimum strain in the formed complexes, thus attaining maximum thermodynamic stability. However, designing and screening that optimal scaffold is very challenging, as even a tiny change of the scaffold structure can produce substantial reduction of the metal affinity (Schwarzenbach, 1952, Cotton and Harris, 1955). For example, the nickel(II)-binding affinity of ethylenediamine is about 5.6 orders of magnitude stronger than that of trimethylenediamine simply due to one methylene variation on the scaffold structure (Cotton and Harris, 1955). Unique approaches are needed to facilitate the discovery of high-affinity metal chelators.

Traditional screening methods require enormous synthetic work to screen a broad palette of scaffolds to identify an optimal scaffold structure for the best fit metal coordination and highest metal affinity, which has proved to be very time consuming and inefficient, especially for designing chelators with complicated structures. For example, enterobactin (ENT), a natural iron chelator primarily produced by *E*. *coli*, is the strongest iron chelator ever known, displaying an iron stability constant of about 10^49^ (Loomis and Raymond, 1991). ENT contains three dihydroxybenzoyl serine groups (ligands) linked in a macrocyclic lactone ring scaffold, which coordinate with Fe(III) to form a hexadentate triscatecholate geometry. The coordination geometry organized by the macrocyclic lactone scaffold and amide linkages contributes to the extraordinarily high iron affinity (Cass et al., 1989, Cohen et al., 1998), which inspired researchers to mimic that unique structure for the development of strong iron chelators by designing various scaffolds (Harris and Raymond, 1979, Harris et al., 1981, Pecoraro et al., 1981, Rodgers et al., 1987, Imbert et al., 2000). However, the iron affinities of these ENT analogs employing various scaffolds obtained by multistep synthesis have not been able to approach the metal affinity of ENT, which is understandable in that any modification or change of that optimal scaffold selected by nature may deleteriously impair the metal-binding affinity.

Besides traditional small molecule chelators, polymer chelators bearing functional ligands on their side chains or backbones have been investigated to take advantage of the properties of macromolecules (Spivakov et al., 1985, Hallaway et al., 1989). In a polymer chelator, a polymer backbone with dynamic conformations can be considered a “soft” scaffold for the conjugated ligands. Thus, soft polymer chelators may coordinate metals and stabilize the metal complexes via unique mechanisms compared with the more rigid scaffolds of small molecular chelators. A flexible polymer chain may reorganize its conformations for the conjugated ligands to coordinate metal ions with the lowest possible strain, thereby promoting the highest possible thermodynamic stability of the formed complexes. Defining functional designs when conjugating ligands to a polymer backbone would provide a simple synthetic and screening approach to potent chelators and avoid complex schemes required when attempting to imitate natural chelators or design new chelators.

Herein, we developed a series of polymeric iron chelators as models and systematically investigated their iron coordination mechanisms to probe the influence of flexible and dynamic macromolecular structure on the metal-binding affinity of the conjugated ligands. We observed significantly enhanced metal-binding affinity of small molecule catechol ligands, increasing by up to 8–9 orders of magnitude after conjugation to a poly(allylamine hydrochloride) (PAH) chain. The enhanced iron coordination resulted from a “conformational stability effect” due to the ability of PAH to spontaneously adopt a specific conformation as an optimal “soft” scaffold for metal coordination with minimum strain in the formed metal complexes and maximum thermodynamic stability. Most importantly, these polymer chelators were prepared by a simple carbodiimide coupling reaction instead of complicated, multistep synthetic routes used in traditional screening methods. Our study also revealed unique coordination characteristics of polymeric iron chelators, highly dependent on their chain flexibility and the strength of metal affinity of the ligands. This study proved that soft polymer chelators with dynamic structures and simple preparation methods could spontaneously achieve the highest possible level of metal affinity of the ligands, even comparable to that of ENT, the strongest iron chelator ever known, thus providing a facile and efficient way of screening strong polymer metal chelators.

## Results

**Figure 1:**
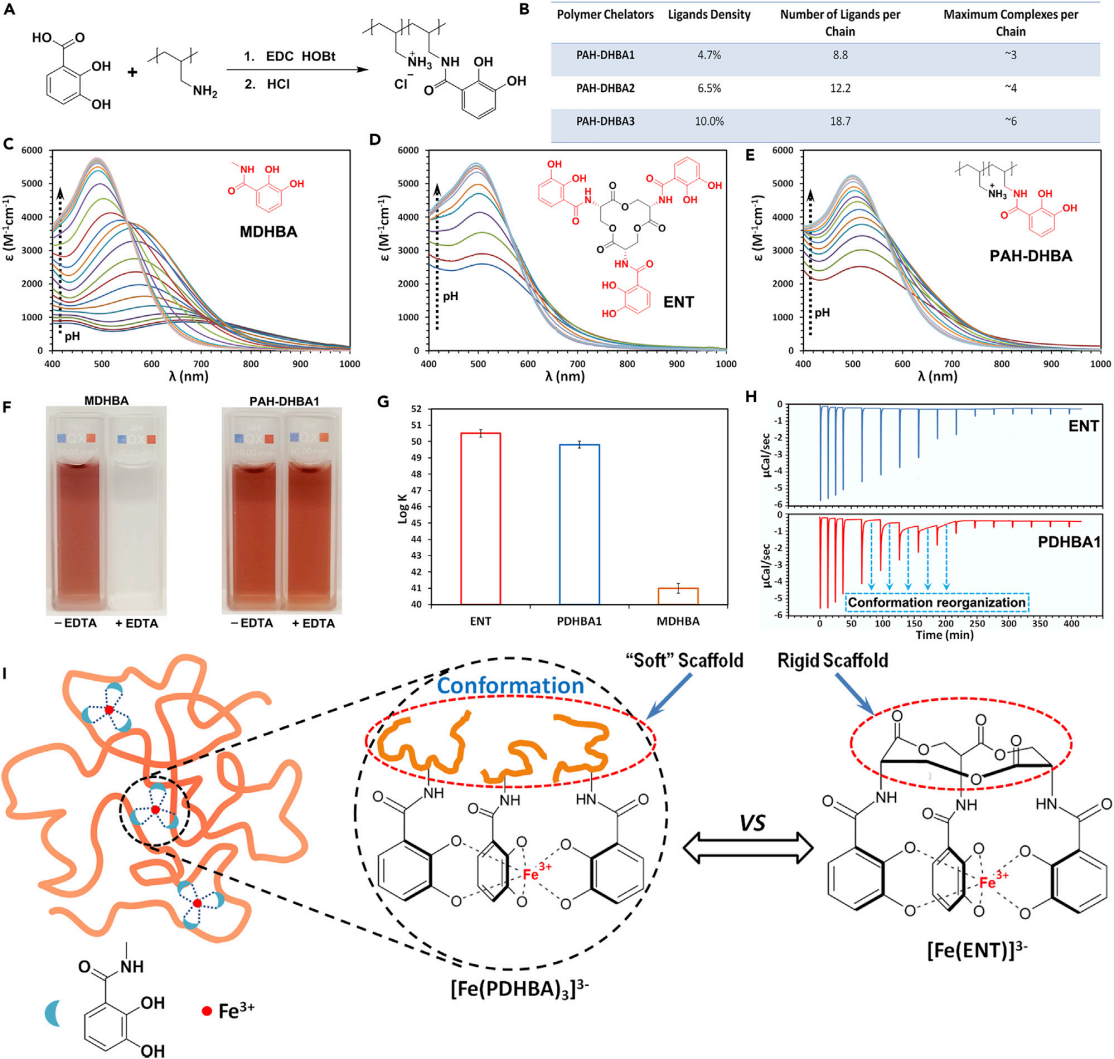
Comparison of Ferric Coordination Characteristics between MDHBA, ENT, and PAH-DHBA (A) Schematic illustration of preparation of PAH-DHBA iron chelators. (B) DHBA ligand density for each PAH-DHBA iron chelator. Ligand density is the molar ratio of conjugated DHBA ligands to total amines of PAH. The number of ligands per polymer chain is calculated based on the ligand density and molecular weight of PAH (molecular weight 17,500 Da, 187 repeat units). The maximum ferric complexes per chain is calculated based on the number of ligands per chain with an iron/ligand ratio of 1/3. (C–E) Visible spectra of ferric MDHBA (C), ENT (D), and PAH-DHBA1 (E) systems as a function of pH. pH ranges: 3.0–9.5 for ferric MDHBA system and 3.25–7.25 for ferric ENT and PAH-DHBA1 systems. (F) Comparison of the iron affinities of MDHBA and PAH-DHBA1 with the competition of EDTA. Molar ratio of Fe: MDHBA (or PDHBA1): EDTA = 1:3:10 at pH 7.0. (G) Iron stability constants of MDHBA, ENT, and PDHBA1 determined by ligand competition assay. Error bars represent the SD of three replicates. (H) Raw ITC curves of ferric ENT (upper panel) and PDHBA1 (lower panel) systems. (I) Schematic illustration of the possible mechanism of conformational stability effect.

Three iron chelators including a small molecule chelator N-methyl-2,3-dihydroxybenzamide (MDHBA), a polymer chelator prepared by conjugating 2, 3-dihydroxybenzoic acid (DHBA) to PAH (PAH-DHBA), and ENT were employed as models to investigate how the macromolecular characteristics affect the affinity of the ligands on the polymer chains (Figures 1C–1E). These molecules all share the same functional ligands (marked red in the structures). Three PAH-DHBA polymers displaying different amounts of DHBA (PAH-DHBA1, PAH-DHBA2, PAH-DHBA3) were prepared by conjugating various amounts of DHBA to PAH chains via a simple carbodiimide coupling reaction (Figure 1A). The DHBA ligands conjugated on the PAH-DHBA1, PAH-DHBA2, and PAH-DHBA3 were termed PDHBA1, PDHBA2, and PDHBA3, respectively, whereas the term “PDHBA” was used to make general reference to all the three types of ligands. The DHBA conjugation was confirmed and analyzed by H^1^ NMR and C^13^ NMR (Figure S1). The molar ratio of PDHBA ligands on PAH-DHBA1, PAH-DHBA2, and PAH-DHBA3 were determined to be 4.7%, 6.5%, and 10.0%, respectively. Based on the molecular weight of PAH (17,500), the average numbers of PDHBA1, PDHBA2, and PDHBA3 ligands on their corresponding polymer chains were calculated to be about 8.8, 12.2, and 18.7, respectively (Figure 1B).

It is well known that ferric catechol complexes exhibit strong and unique pH dependency. As the pH increases, catechols form mono-, bis-, and then tris-complexes with ferric ions (Avdeef et al., 1978, Sever and Wilker, 2004). Ferric MDHBA complexes showed typical pH dependency commensurate with other ferric catechol systems. Figure 1C is the visible spectrum of the ferric MDHBA system as a function of pH between 3.0 and 9.5. Three distinct peaks appearing with λ_max_ around 490, 570, and 740 nm corresponded to the visible absorption of Fe(MDHBA)_3_, Fe(MDHBA)_2_, and FeMDHBA complexes, respectively. The spectra also showed two isosbestic points, around 550 nm between pH 6.0 and 9.0, and around 710 nm between pH 4.0 and 6.0. The first isosbestic point corresponded to the equilibrium between Fe(MDHBA)_3_ and Fe(MDHBA)_2_, whereas the second one corresponded to another equilibrium between Fe(MDHBA)_2_ and FeMDHBA (Harris et al., 1979a). Each equilibrium results from a single, two-proton step to add or lose a whole MDHBA ligand (Figure S8).

Figure 1D shows the visible spectra of the ferric ENT system as a function of pH between 3.25 and 7.25. The FeENT complex showed a unique pH dependency different from that of ferric catechol systems as already investigated by other research groups (Harris et al., 1979a, Loomis and Raymond, 1991). The visible spectrum of ferric ENT did not change above pH 7.0, which indicated that the [Fe(ENT)]^3-^ complex is fully formed at this pH. As the pH decreased from 7.0 to 4.0, one sharp isosbestic point appeared around 560 nm, which indicated that only one simple equilibrium between two ferric complexes existed in this condition. As opposed to the ferric MDHBA (or other catechol) system, the protonation of ferric ENT complex showed a one-proton step instead of the dissociation of a whole DHBA ligand (two-proton step) upon acidification, which was described by the equation derived by Schwarzenbach (Harris et al., 1979a). Protonation with a one-proton step is indicative of the high stability of the [Fe(ENT)]^3-^ complex. The strong iron affinity of ENT primarily originates from its unique tri-L-serine lactone backbone and the amide linkages, which pre-organize the three catecholate ligands to facilitate iron coordination and stabilize the complexes (Stack et al., 1992).

**Figure 2:**
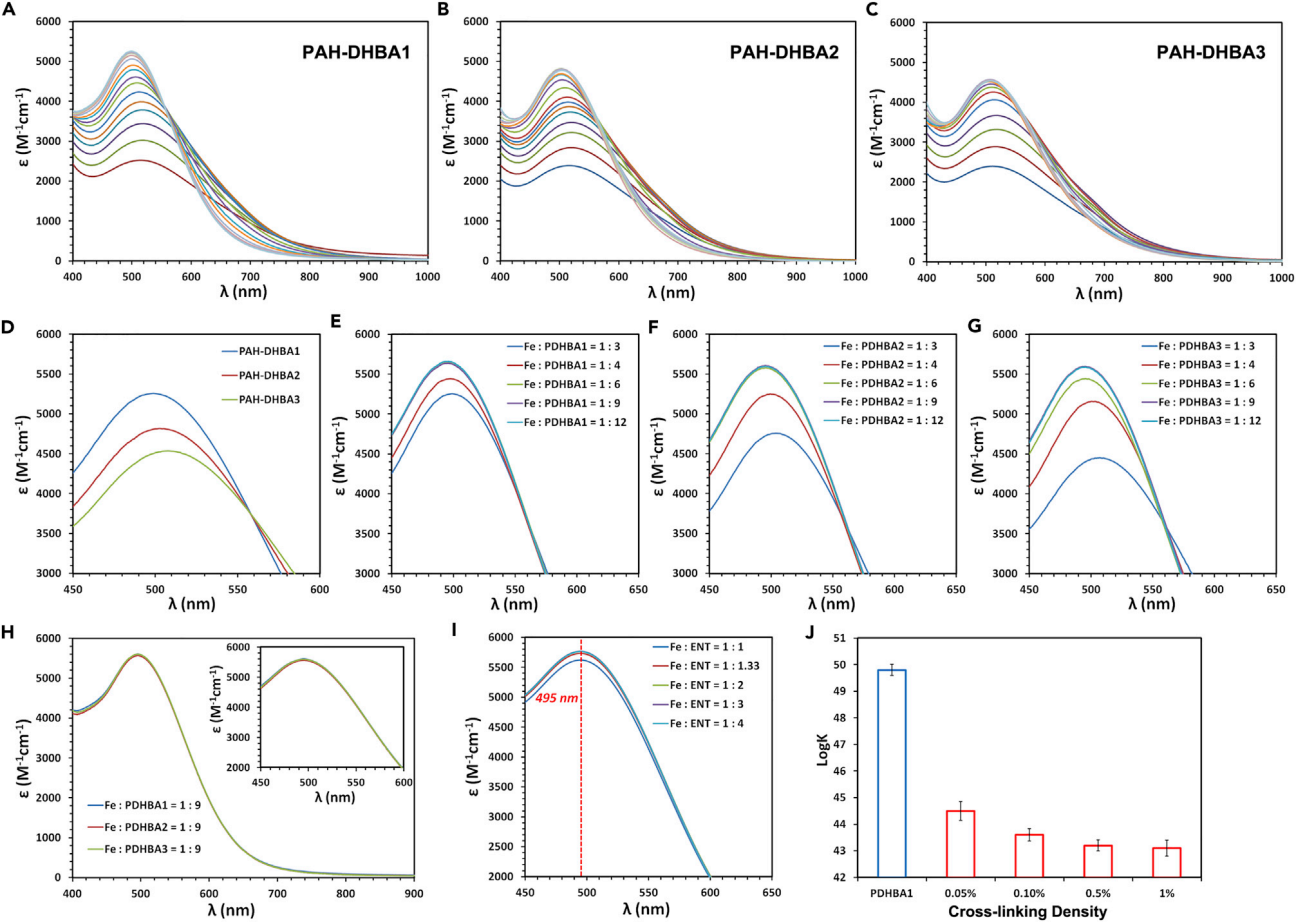
Chain Flexibility of Polymer Chelators can Influence the Iron Chelation Characteristics (A–C) Visible spectra of ferric PAH-DHBA1 (A), PAH-DHBA2 (B), and PAH-DHBA3 (C) systems as a function of pH. pH ranges: 3.25–7.25. (D) Visible spectra of ferric PAH-DHBA polymers with various PDHBA contents at pH 7.0. Condition: 0.05 mM Fe^3+^, 0.15 mM PDHBA1 (or PDHBA2, PDHBA3) in 1 M KCl solution. (E–G) Visible spectra of ferric PAH-DHBA1 (E), PAH-DHBA2 (F), and PAH-DHBA3 (G) systems with various Fe/PDHBA molar ratios at pH 7.0. Condition: 0.05 mM Fe^3+^ in 1 M KCl solution. (H) Visible spectra of PAH-DHBA1, PAH-DHBA2, and PAH-DHBA3 systems at Fe/PDHBA molar ratio of 1/9. Condition: 0.05 mM Fe^3+^, 0.45 mM PDHBA1 (or PDHBA2, PDHBA3) in 1 M KCl solution at pH 7.0. (I) Visible spectra of ferric ENT system with various Fe/ENT molar ratios at pH 7.0. The ENT/Fe ratios were 1, 1.33, 2, 3, and 4, which corresponded to the PDHBA/Fe ratios of 3, 4, 6, 9, and 12 in the ferric PDHBA systems, respectively. Condition: 0.02 mM Fe^3+^ in 1 M KCl solution. (J) Iron stability constants of PAH-DHBA1 with various cross-linking densities. Error bars represent the SD of three replicates. Cross-linking density is the molar ratio of cross-linker (N,N-methylene bisacrylamide) to total amines of PAH-DHBA1 polymer.

The PAH-DHBA polymer chelators shared the same functional groups with MDHBA and ENT. However, as these PDHBA ligands were randomly distributed on the polymer backbones, there are no predisposed structures organized by a rigid scaffold like the macrocyclic lactone ring of ENT facilitating iron coordination. Hence, the coordination characteristics of PDHBA ligands were anticipated to be closer to MDHBA, but not ENT. The PDHBA ligands, however, showed surprisingly similar ferric coordination characteristics with ENT instead of MDHBA (Figure 1E). Like the ferric ENT system, the visible spectrum of the ferric PDHBA1 system did not change above pH 7.0 either. As the pH decreased from 7.0 to 4.0, a single, sharp isosbestic point also appeared around 560 nm, which indicated that only one simple equilibrium between two ferric complexes existed in this condition. Moreover, the ferric PDHBA1 complex also showed a one-proton step in the protonation process instead of the dissociation of a whole PDHBA ligand, which was also confirmed by the Schwarzenbach equation (Figure S10A). Consequently, there was only [Fe(PDHBA1)_3_]^3-^ and a less stable [FeH(PDHBA1)_3_]^2-^ complex equilibrating in solution at that pH region. The pH dependency of the ferric complexes prepared from PAH-DHBA2 and PAH-DHBA3 polymers was also investigated. All three ferric complexes exhibited similar pH-dependent visible spectra with no changes above pH 7.0 and sharp isosbestic points around 560 nm (Figures 2A–2C). The only differences were the wavelength of maximum absorbance (λ_max_) and the values of maximum absorbance recorded at the same pH, differences that will be discussed in more detail below.

The distinct differences in the iron coordination processes of PDHBA and MDHBA suggested significant differences in iron affinity. With the competition of EDTA, the absorption of the ferric MDHBA complexes dramatically decreased at pH 7.0 and totally disappeared at pH 6.0 (Figure S9). However, the absorption of the ferric PDHBA1 complexes did not change at all at pH 6.0 and 7.0. Even with a 10-fold excess EDTA concentration, no distinguishable changes in the spectra were observed (Figures 1F and S9). These results clearly indicated that PDHBA1 possessed superior iron affinity compared with MDHBA. The iron stability constants of the three chelators were determined by a ligand competition assay (Harris et al., 1979b). The iron stability constants of MDHBA, ENT, and PDHBA1 were determined to be 41.1, 50.5, and 49.7, respectively (Figure 1G). Surprisingly, PDHBA1 showed more than 8 orders of magnitude enhancement in iron affinity compared with its small molecule counterpart MDHBA, and similar to the most powerful iron chelator, ENT. This result supports a design concept using simple conjugation of small molecule ligands to flexible polymer backbones to produce a substantial enhancement in metal-binding affinity.

It should be emphasized that the concentrations of the polymer chelators selected for all the studies were all below 0.03 wt. %, which is well below the critical overlap concentration of PAH (c* ∼2.5 wt. %) (Chollakup et al., 2010). Consequently, any inter-polymer ferric PDHBA complexation should be negligible, and thus metal complexation and transformation between different ferric PDHBA complexes should mainly take place on each single polymer chain (intra-polymer). In addition, the formation of [Fe(PDHBA)_2_]^-^ and especially [Fe(PDHBA)_3_]^3-^ complexes requires effective conformational changes of each polymer chain to allow the engagement of the randomly distributed PDHBA ligands and ferric ions. Such processes would have to overcome energy barriers, which were speculated to impair the iron affinity of PDHBA. On the contrary, the PDHBA ligands substantially enhanced iron affinity via participation of the polymeric structure. The flexible polymer chain may spontaneously adopt a specific conformation as an optimal “soft” scaffold to assist the conjugated PDHBA ligands in stabilizing ferric ions in the best fit coordination geometry that could produce the lowest possible strain in the formed complexes with the highest possible thermodynamic stability. That best fit coordination geometry organized by that specific conformation might be very similar to the coordination geometry of ferric ENT, which might be the best ferric coordination geometry selected by microorganisms for catechol-type ligands.

To adopt that optimal conformation, the polymer chain has to continuously reorganize its conformation during the coordination process until achieving the highest thermodynamic stability of the system. This chain conformation reorganization process could be observed by isothermal titration calorimetric (ITC) analysis. As shown in Figure 1H, the ITC curve of Fe-PDHBA1 system is distinctly different from that of Fe-ENT system, which exhibited a typical ITC curve with normal exothermic peaks attributed to the heat from the iron-ENT coordination reaction process. In contrast, for the Fe-PDHBA1 system, except the first four injections with normal peaks, the following injections produced exothermic peaks with remarkable tails, which indicated that a slow kinetic exothermic process existed after the iron-PDHBA coordination reaction process. This unique process should be attributed to the relatively slow chain conformation reorganization (compared with the fast iron coordination reaction) for progressively seeking the most stable coordination geometry (the lowest strain in the formed complexes, i.e., the lowest possible energy state) with continuous heat generation. The ITC data clearly supported our hypothesis that the polymer chain could spontaneously seek and adopt optimal conformations for the most stable metal coordination. Achieving this conformation as a “soft” scaffold may, therefore, mimic the geometry of the rigid macrocyclic lactone scaffold of ENT to facilitate iron coordination and stabilize the formed complexes (as illustrated in Figure 1I). The substantial additional stability originating from the induced specific polymer conformation was dubbed the “conformational stability effect.” Based on this hypothesis, any restriction of the chain flexibility could potentially hinder the formation of the optimal conformation and diminish the ability to form the most stable [Fe(PDHBA)_3_]^3-^ complexes.

PDHBA density on the PAH-DHBA chain, or in other words, the number of ferric complexes formed per chain, is a critical factor that could influence the chain flexibility during the formation of the complexes. The formation of one [Fe(PDHBA)_3_]^3-^ complex on one PAH-DHBA polymer chain may be considered analogously to an intra-molecular cross-linking, which leads to restriction of its flexibility. Hence, the first several [Fe(PDHBA)_3_]^3-^ complexes with the optimal conformation can be formed on a single polymer chain until the chain flexibility begins to be restricted. Subsequent formation of complexes may become more difficult to adopt the optimum conformation (scaffold) due to the restriction of chain flexibility by these previously formed complexes. Consequently, it would be anticipated that, with the same PDHBA/Fe molar ratio (3/1), higher PDHBA density would lead to more complexes with lower stability formed on each polymer chain. This speculation was confirmed by further analyzing the visible spectra of the three ferric PDHBA systems. Figure 2D showed the visible absorption maxima of the complexes prepared from the three PAH-DHBA polymers with a fixed Fe/PDHBA molar ratio (1/3) at pH 7.0. The spectra clearly showed that increasing PDHBA density led to the red shift of λ_max_ and reduction of absorbance. The visible spectra of PAH-DHBA1, PAH-DHBA2, and PAH-DHBA3 showed λ_max_ of 497, 503, and 507 nm, respectively. Interestingly, the spectra also showed one sharp isosbestic point appearing around 560 nm, which is coincident with the one observed in the visible spectra with pH changing. The isosbestic point indicated that only one simple transformation between two ferric complexes existed as the PDHBA density was varied at the same pH (7.0).

As already concluded above, only [Fe(PDHBA)_3_]^3-^ and [FeH(PDHBA)_3_]^2-^ existed in solution at this pH, and [FeH(PDHBA)_3_]^2-^ had a longer λ_max_ and lower molar extinction coefficient (ϵ) compared with [Fe(PDHBA)_3_]^3-^. Hence, the red shift of λ_max_ and reduction of ϵ indicated more [FeH(PDHBA)_3_]^2-^ with lower stability formed as PDHBA density increased. These results supported our speculation that restriction of polymer chain flexibility could lower the probability to adopt an optimal conformation for the most stable [Fe(PDHBA)_3_]^3-^ complexes and lead to the formation of less stable [FeH(PDHBA)_3_]^2-^ complexes. Preservation of the exact same isosbestic point indicated that changing pH or PDHBA density may equally contribute to the equilibrium between [Fe(PDHBA)_3_]^3-^ and [FeH(PDHBA)_3_]^2-^ complexes. It should be pointed out that, the ligand density should not be too low, or the polymer chain would start to hinder the iron coordination process due to the low chance of engaging three PDHBA ligands on a single polymer chain (Figure S13).

The ligand to metal (PDHBA/Fe) ratio is another factor that could influence the chain flexibility during the formation of complexes. It should be noted that even the ferric PDHBA1 system showed a longer λ_max_ (497 nm) compared with the ferric ENT system (495 nm), which indicated that even the PAH-DHBA1 polymer chain with the lowest ligand density was restricted during formation of complexes at the PDHBA/Fe ratio of 3/1. Thus, increasing the PDHBA/Fe ratio could decrease the number of complexes formed per polymer chain and provide more flexibility to adopt the most favorable conformation. Increasing the PDHBA/Fe ratio may then lead to a blueshift of the λ_max_ and increased absorbance. Figures 2E–2G showed the visible absorption maxima of the ferric PDHBA1, PDHBA2, and PDHBA3 systems with various PDHBA/Fe molar ratios at pH 7.0, respectively. As PDHBA/Fe molar ratio increased from 3 to 12, blueshifts of the λ_max_ from 497 to 495 nm, 502 to 495 nm, and 506 to 495 nm were clearly observed for ferric PDHBA1, PDHBA2, and PDHBA3 systems, respectively. Besides the blueshifts of all three λ_max_ to a fixed wavelength (495 nm), the ϵ of the three systems also increased to a fixed value of 5,650 M^−1^cm^−1^, which indicated that the [Fe(PDHBA)_3_]^3-^ complex was fully formed in every ferric PDHBA system at high PDHBA/Fe molar ratios (9 and 12). Actually, the three systems showed exactly identical visible spectra when the PDHBA/Fe molar ratio was above 9 (Figure 2H).

The identical spectra at high PDHBA/Fe ratios indicated that the three ferric PDHBA systems could exhibit the same ferric coordination characteristics by reducing the number of complexes per chain (i.e., providing sufficient chain flexibility). λ_max_ (495 nm) and ϵ (5650 M^−1^cm^−1^) were the two characteristic values of the [Fe(PDHBA)_3_]^3-^ complexes. Coincidently, the two critical characteristic values were almost identical with [Fe(ENT)]^3-^ complexes (λ_max_ 495 nm, ϵ 5,700 M^−1^cm^−1^), which indicated that the most favorable conformation adopted by the [Fe(PDHBA)_3_]^3-^ complexes may accurately mimic the coordination geometry of [Fe(ENT)]^3-^ complexes. These results could also explain why PDHBA ligands earned comparable iron affinity with ENT. Like the visible spectra of changing PDHBA density, all the three spectra generally showed the same isosbestic point close to 560 nm, which indicated that all three systems also exhibited only one identical transformation between two identical ferric complexes [Fe(PDHBA)_3_]^3-^ and [FeH(PDHBA)_3_]^2-^ with the change of Fe/PDHBA ratios.

The influence of the ENT/Fe ratio was also investigated as a comparison with ferric PDHBA systems (Figure 2I). As opposed to PDHBA, only a slight increase of absorbance was observed as the ENT/Fe ratio increased from 1 to 1.33, and no further remarkable increase was observed as the ratio increased. Most importantly, the λ_max_ was invariant (495 nm) and no isosbestic point was observed, which indicated that no transformation between two ferric ENT complexes ([Fe(ENT)]^3-^ and [FeH(ENT)]^2-^ occurred with the alternation of ENT/Fe ratio. This result implied that changing the ENT/Fe ratio could not alter the ferric complexation characteristics of ENT. The dramatic difference between ferric PDHBA and ENT systems implied that the macromolecular characteristics of polymer chelators, such as chain flexibility and conformation, provide a unique metal coordination characteristic, including an induced conformational stability effect.

Other factors such as the molecular weight of PAH can also influence the coordination behavior. With the same ligand modification molar ratio, higher-molecular-weight polymer has more ligands per polymer chain and would form a larger number of less-stable [FeH(ENT)]^2-^ complexes compared with the lower-molecular-weight polymer (Figure S12). The original flexibility of the polymer chain can also influence the metal coordination behavior. For example, DHBA-modified linear polyethyleneimine (L-PEI-DHBA) can form more stable complexes than DHBA-modified branched polyethyleneimine (B-PEI-DHBA), likely due to the higher chain flexibility of L-PEI than B-PEI (Figure S11).

It is well known that the [Fe(ENT)]^3-^ complex adopts a Δ-configuration at the iron center (Bluhm et al., 2002). The [Fe(PDHBA)_3_]^3-^ complex formed a racemic mixture of both Λ and Δ enantiomers indicated by circular dichroism spectroscopy (Figure S10B), which is reasonable because the PAH-DHBA polymers did not contain any chiral structures. This result also indicated that the Λ enantiomer of [Fe(PDHBA)_3_]^3-^ complex should have a similar iron affinity with the Δ enantiomer and the configuration is not a key factor affecting iron affinity.

Direct chemical cross-linking of PAH-DHBA polymer chains is another more effective way of restricting the chain flexibility. After cross-linking by N,N-methylene bisacrylamide, the polymer chains were restricted in the polymer network, thus adoption of optimal conformation for ferric coordination was highly constrained. When the polymer chains were cross-linked even with a low cross-linking density (0.05%), the iron stability constant decreased by more than 5 orders of magnitude. Further cross-linking correspondingly decreased the stability constant but with diminishing effects (Figure 2J).

**Figure 3:**
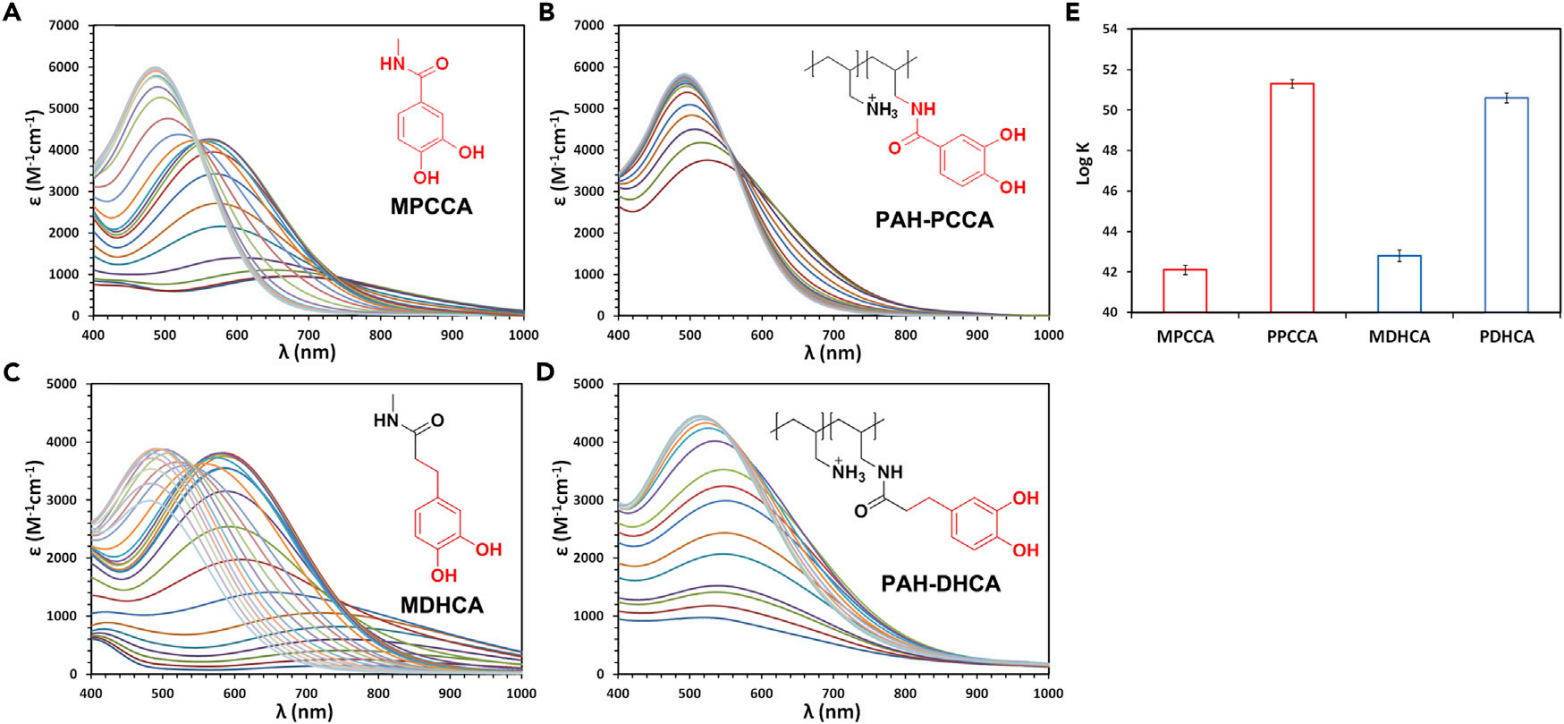
Conformational Stability Effect Can Be Observed on Other Polymer Catechol Chelators (A and B) Visible spectra of ferric MPCCA (A) and PAH-PCCA (B) systems as a function of pH. pH ranges: 4.0–9.5 for ferric MPCCA system and 4.2–8.5 for ferric PAH-PCCA system. (C and D) Visible spectra of ferric MDHCA (C) and PAH-DHCA (D) systems as a function of pH. pH ranges: 4.0–10.0 for ferric MDHCA system and 4.0–8.5 for ferric PAH-DHCA system. Condition: 0.05 mM Fe^3+^, 0.15 mM ligands in 1 M KCl solution. (E) Iron stability constants of MPCCA, PPCCA, MDHCA, and PDHCA determined by ligand competition assay. Error bars represent the SD of three replicates.

The “conformational stability effect” was probed using different metal ligands on polymers. Two other catechols, 3,4-dihydroxybenzoic acid (protocatechuic acid, PCCA) and 3,4-dihydroxyhydrocinnamic acid (DHCA), were employed to prepare PAH-PCCA and PAH-DHCA polymer chelators (Figures S2 and S3). As shown in Figures 3B and 3D, both PPCCA and PDHCA showed very similar patterns of pH dependency with PDHBA and ENT. In contrast, both their small molecule counterparts, N-methyl-3,4-dihydroxybenzoic acid (MPCCA) and N-methyl-3,4-dihydroxyhydrocinnamamide (MDHCA), showed typical pH dependency pattern of catechols (Figures 3A and 3C). One exception is that MDHCA showed a remarkable reduction of absorbance at high pH (above 8.0) possibly due to oxidation. The similar ferric coordination characteristics of the two polymer chelators with PDHBA and ENT indicated that the conformational stability effect may also apply to the PPCCA and PDHCA ligands. The iron stability constants determined by the ligand competition assay confirmed that PPCCA and PDHCA exhibited 9 and 8 orders of magnitude enhancement, respectively, compared with their small molecule counterparts MPCCA and MDHCA, respectively (Figure 3E). The two polymers may also adopt optimal conformations for the PPCCA and PDHCA ligands to form ferric complexes with the lowest strains and highest thermodynamic stabilities. Based on these data, we might speculate that other catechols could also acquire the conformational stability effect once conjugated to PAH.

The conformational stability effect could take effect not only in ferric but also in other metal polymer chelator systems. Titanium(IV) was reported to coordinate with catechols to form stable complexes with a 1:3 stoichiometry (Borgias et al., 1984). pH-dependent studies of Ti(IV)-MDHBA and Ti(IV)-PDHBA1 complex systems showed that the Ti(IV)-PDHBA1 complexes are extremely stable as the pH decreases (competition from protons) compared with Ti(IV)-MDHBA complexes (Figure S14A and S14B). At pH 2.0, the Ti(IV)-MDHBA complexes were fully disassociated as the absorption peak totally disappeared. However, above pH 2.0, the spectrum of Ti(IV)-PDHBA1 system did not change, indicating full formation of [Ti(PDHBA1)_3_]^2+^ complexes at this pH. With the competition of EDTA, the absorption of the Ti(IV)-MDHBA complexes dramatically decreased at pH 4.0 and totally disappeared at pH 3.0 (Figure S14C and 14D). However, the absorption of the Ti(IV)-PDHBA1 complexes did not change at all, even at pH 1.0 (Figure S14E). Only with the competition of pentetic acid (DTPA), a stronger chelator than EDTA, the absorption of the Ti(IV)-PDHBA1 complexes started to decrease (Figure S14F). These data clearly indicated that the Ti(IV) affinity of PDHBA ligands was substantially enhanced (more than 8 orders of magnitude) compared with the small molecule counterpart MDHBA.

**Figure 4:**
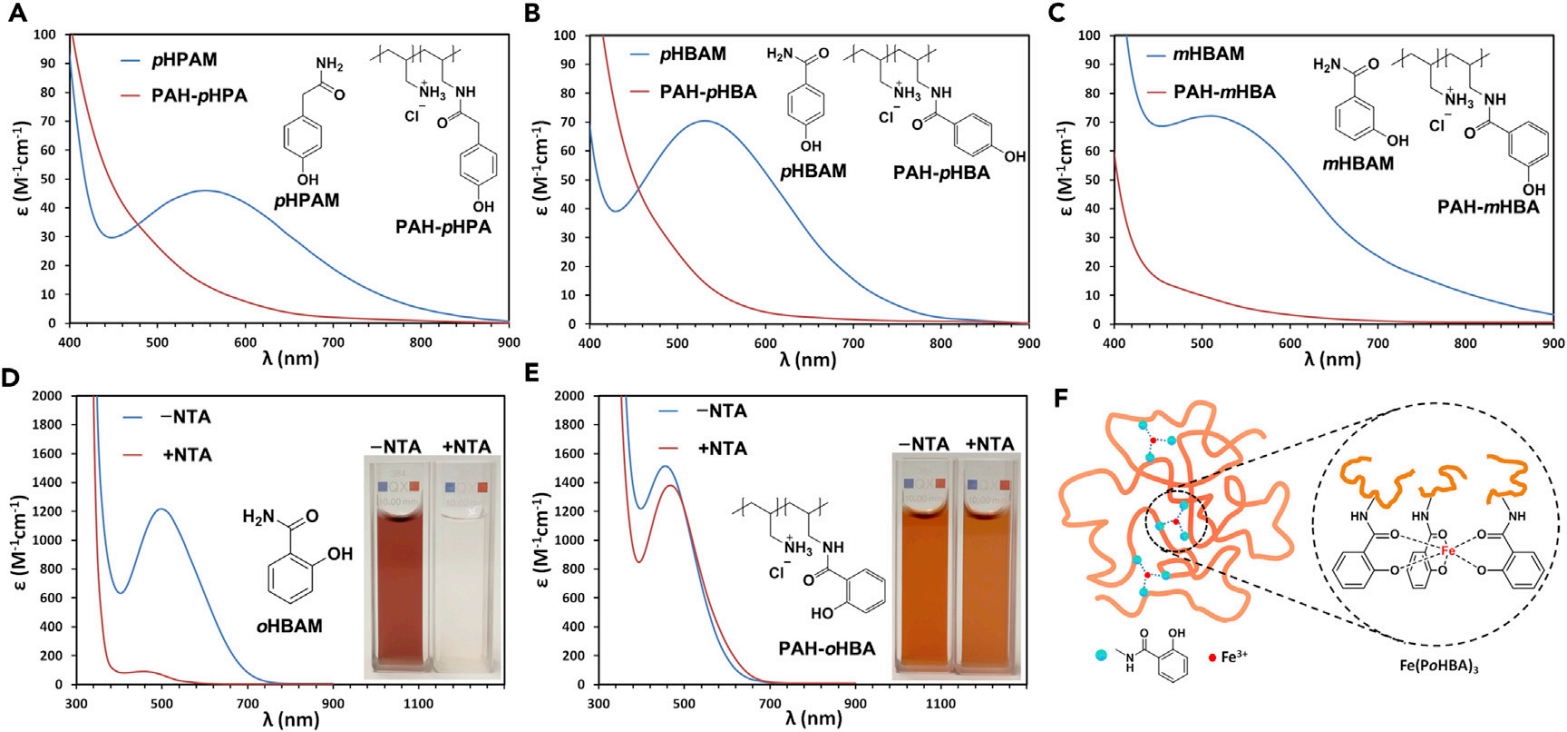
Weak Monodentate Ligands on Polymer Chains Cannot Induce Conformational Stability Effect (A) Comparison of visible spectra of ferric *p*HPAM and PAH-*p*HPA systems. Condition: 1 mM Fe^3+^, 4 mM ligands in 0.1 M KCl solution at pH 2.6. (B) Comparison of visible spectra of ferric *p*HBAM and PAH-*p*HBA systems. Condition: 1 mM Fe^3+^, 4 mM ligands in 0.1 M KCl solution at pH 2.6. (C) Comparison of visible spectra of ferric *m*HBAM and PAH-*m*HBA systems. Condition: 1 mM Fe^3+^, 4 mM ligands in 0.1 M KCl solution at pH 2.6. (D) Visible spectra of ferric *o*HBAM system with the competition of NTA. Condition: 1 mM Fe^3+^, 4 mM ligand, and 1 mM NTA in 0.1 M KCl solution at pH 4.0. (E) Visible spectra of ferric PAH-*o*HBA system with the competition of NTA. Condition: 1 mM Fe^3+^, 4 mM ligand, and 1 mM NTA in 0.1 M KCl solution at pH 4.0. (F) Schematic illustration of ferric coordination with PAH-*o*HBA.

Not all ligands conjugated to polymers would be expected to induce a conformational stability effect. To form stable complexes, the ligand should possess sufficient metal affinity to counteract the line tension from the dynamic polymer chain during the sequential coordination process. This phenomenon suggests that the coordination process of polymer ligands may have higher energy barriers (activation energy) than that of small molecule ligands. The catechol ligands should be strong enough to overcome such barriers to form stable ferric complexes as confirmed by the above studies. It is necessary to investigate the coordination characteristics of polymer chelators with weaker ferric ligands compared with catechol, such as phenols. Phenols are weak monodentate ligands and coordinate with ferric iron with a 3:1 stoichiometry in the pH range 1.7–3.0 (Moeller and Shellman, 1953). Herein, *p*-hydroxyphenylacetic acid (*p*HPA) and three substitution isomers, *p*-hydroxybenzoic acid (*p*HBA), *m*-hydroxybenzoic acid (*m*HBA), and *o*-hydroxybenzoic acid (*o*HBA) were employed to prepared PAH-*p*HPA, PAH-*p*HBA, PAH-*m*HBA, and PAH-*o*HBA polymeric chelators, respectively (Figures S4–S7). Surprisingly, except *o*HBA, all phenol ligands lost iron chelation capabilities after conjugation to PAH, because no distinct peaks of the anticipated complexes were observed compared with their small molecule counterparts, *p*-hydroxyphenylacetamide (*p*HPAM), p-hydroxybenzamide (*p*HBAM), and m-hydroxybenzamide (*m*HBAM) (Figures 4A–4C and S15).

We speculate that iron coordination with monodentate ligands is too weak and cannot counteract the line tension from the polymer chain to overcome the energy barrier. Hence, polymeric chelators may totally deprive low-affinity monodentate ligands of their metal chelation capabilities. Interestingly, PAH-*o*HBA polymer was an exception. Owing to the special position of the phenolic hydroxyl group, it can cooperate with its neighboring carbonyl group to be a bidentate ligand for ferric coordination (Figure 4F). This salicylamide coordination model was reported previously (Cohen et al., 1998). As shown in Figures 4D and 4E, with the competition of nitrilotriacetic acid (NTA), PAH-*o*HBA exhibited much stronger iron affinity compared with its small molecular counterpart *o*HBAM. These bidentate salicylamide ligands are strong enough to counteract the tension from the polymer chains during the sequential coordination process, so only PAH-*o*HBA polymer can induce the conformational stability effect (5 orders of magnitude enhancement of iron affinity) for iron chelation compared with other monodentate phenol ligands. These data clearly indicate that only the ligands (usually bidentate or polydentate) with certain level of metal affinities can induce the conformational stability effect once conjugated on polymer chains. Surprisingly, the metal ligands on polymer chains act dichotomously depending on their strength of metal affinities: strong chelators can become substantially stronger, whereas weak chelators can become substantially weaker.

The conformational stability effect reported here should not be confused with another class of polymeric chelators in which each individual ligand on the polymer chain can solely coordinate with the metal ion. Such ligands can neither obtain the conformational stability effect nor substantially lose metal affinity as the polymer chain does not participate in the coordination and no specific conformational change is required for the formation of complexes. In such cases, the ligands should have approximately the same metal affinities before and after conjugation to polymers, which has been confirmed by the data from several research groups (Zhou et al., 2006, Zhou et al., 2008, Zhou et al., 2015). Actually, such polymer chelators were among the most investigated polymeric chelators during the past half century, which may be the reason that this conformational stability effect was not observed.

The conformational stability effect may have direct impact on another type of polymer metal chelators, metalloproteins (Lu et al., 2009). In many metalloproteins, metal ions are coordinated by the ligands belonging to amino acid residues of the metalloprotein. However, a much larger amount of these amino acids or other even stronger ligands also exist as either free amino acids or within other peptides or proteins. How can these metal centers maintain enough stability for biochemical activities with extensive competition from a huge concentration of competing ligands, or in other words, why do ligands located in the metal center have a relatively superior metal affinity to competitors? We speculate that the coordination geometry of the metal center organized by the specific conformation adopted by the metalloprotein leads to additional stability of the metal center, which may be the same effect observed in our ferric-polymer chelator systems. The only difference is that, the conformations adopted by metalloproteins may be relatively static due to the three levels of protein structure, whereas the conformations adopted by our polymer chelators are likely to be more dynamic. Moreover, the more rigid structures and fixed conformations of proteins produce pre-organized metal-binding sites with lowered line tension from the polypeptide backbones compared with unstructured, dynamic polymer chains. All these factors can facilitate the coordination process by lowering the activation energy to obtain the substantial additional change of Gibbs free energy resulting from the conformational stability effect.

## Discussion

In summary, we demonstrate a “conformational stability effect” that can significantly enhance the metal affinity of catechol ligands after conjugation to a PAH chain. A flexible polymer chain can spontaneously adopt a specific conformation as an optimal “soft” scaffold for the ligands to coordinate ferric ions with the lowest strain and the highest thermodynamic stability of the formed complexes. Our study also reveals unique coordination characteristics of polymeric iron chelators, which were highly dependent on their chain flexibility and the strength of metal affinity of conjugated ligands. This study demonstrates that soft polymer chelators may spontaneously produce the highest possible metal affinities of the conjugated ligands by reorganizing the conformations, which could obviate the work required to synthesize traditional rigid scaffolds. The conformational stability effect described here proposes a new approach to design metal chelators with extremely high metal affinities that could be extrapolated to many applications. Moreover, the simple coupling reaction method provides a simple and efficient way of developing and screening metal chelators.

### Limitations of the Study

In this study, the “conformational stability effect” was only demonstrated for polyallylamine polymers modified with catechols. Other types of metal ligands and polymer backbones need to be tested to confirm the broader applicability of this effect.

## Methods

All methods can be found in the accompanying Transparent Methods supplemental file.

## Supplemental Information

Document S1. Transparent Methods and Figures S1–S15
