# Supplementary material for: Conformational Stability Effect of Polymeric Iron Chelators
Source: iScience. 2019 Oct 14;21:124–34. doi: 10.1016/j.isci.2019.10.022 (PMC6820273; doi:10.1016/j.isci.2019.10.022)
Supplement: Document S1. Transparent Methods and Figures S1–S15 [file mmc1.pdf]

ISCI, Volume *21*

Supplemental Information

Conformational Stability Effect of Polymeric Iron Chelators

Jian Qian and Cory Berkland

Supplemental figures

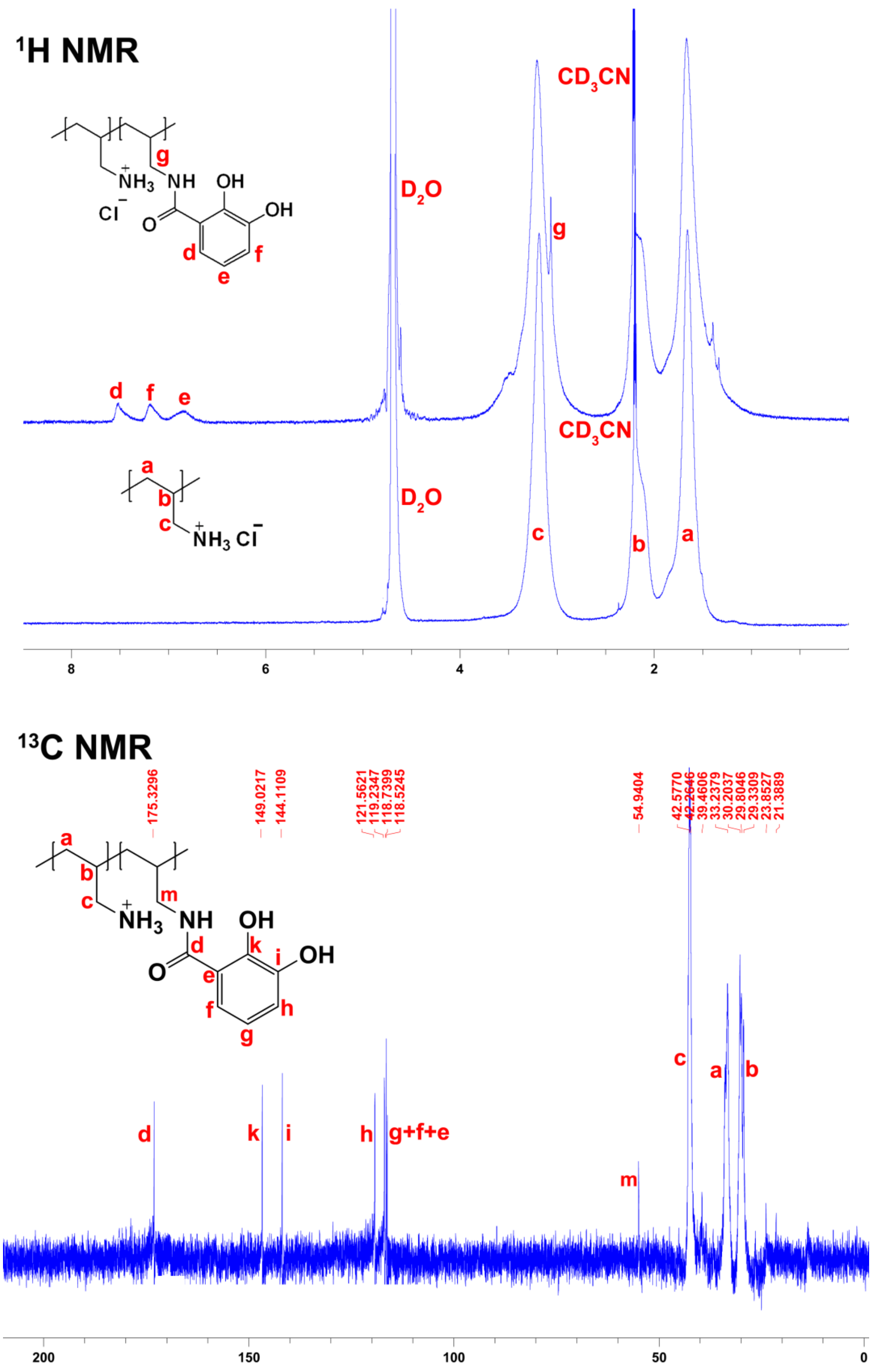

Figure S1. ^{1}H NMR and ^{13}C NMR of PAH-DHBA1 polymer, related to Figure 1.

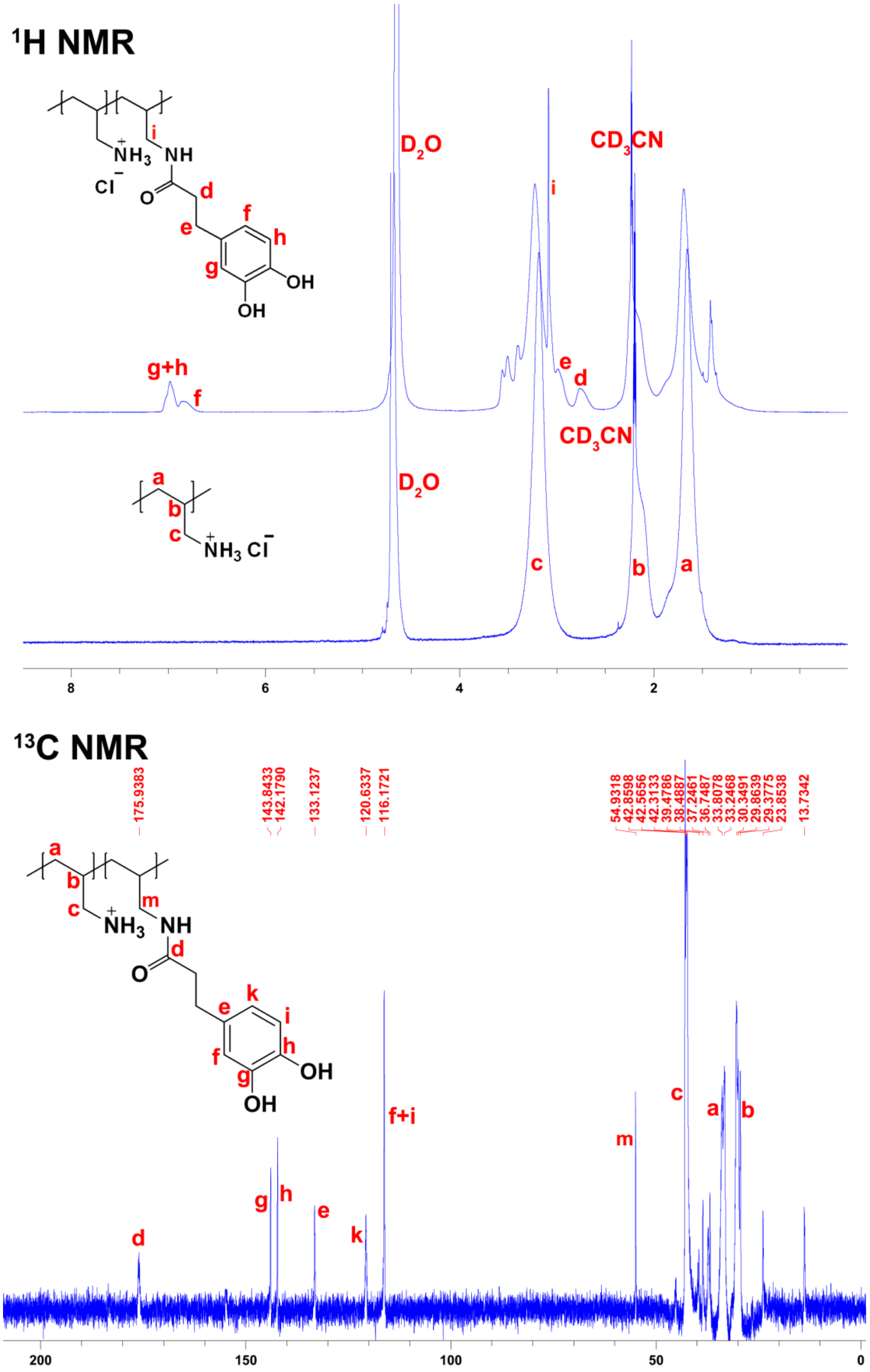

Figure S2. ^{1}H NMR and ^{13}C NMR of PAH-DHCA polymer, related to Figure 1.

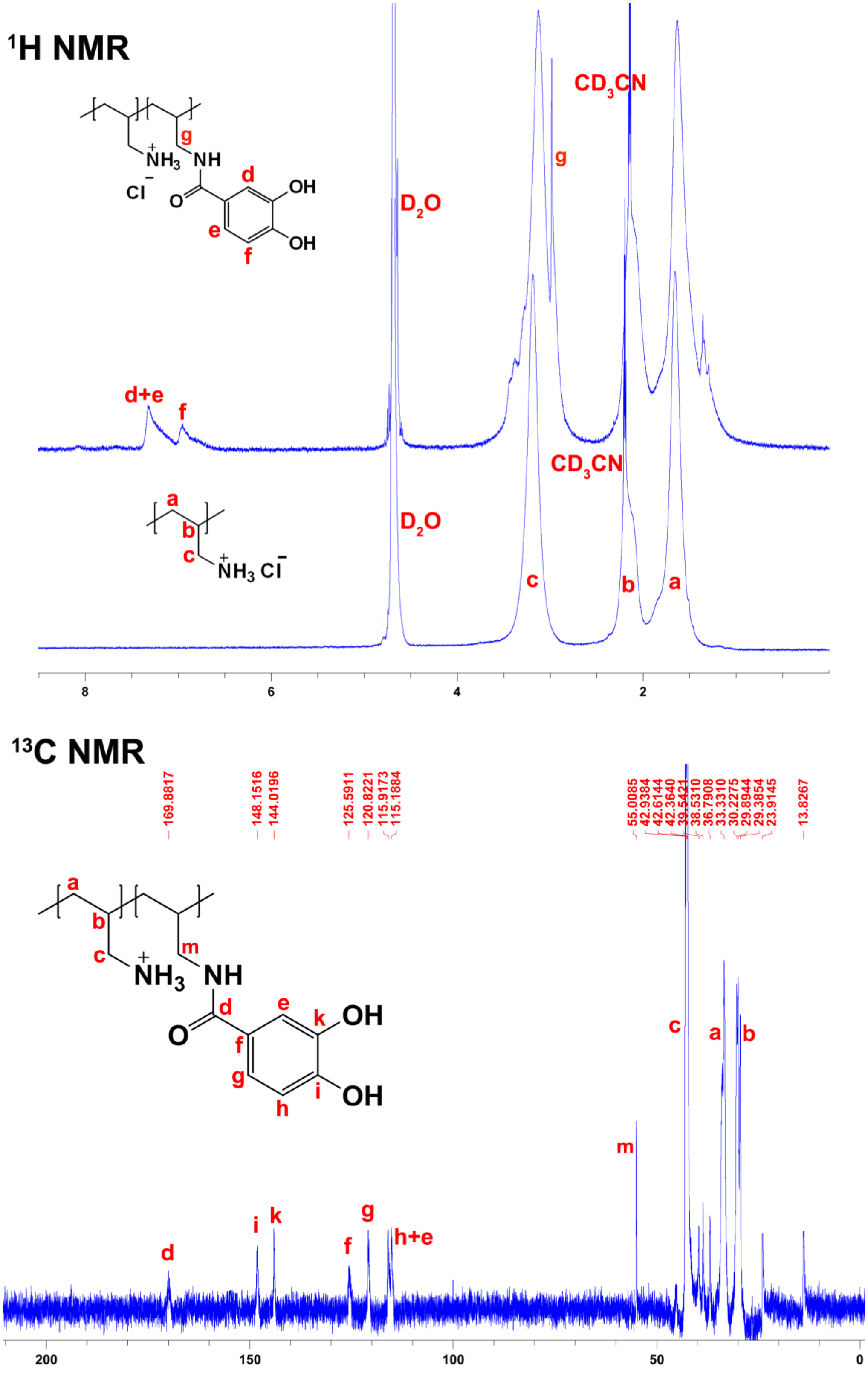

Figure S3. ^{1}H **NMR and** ^{13}C **NMR of PAH-PCCA polymer, related to Figure 1.**

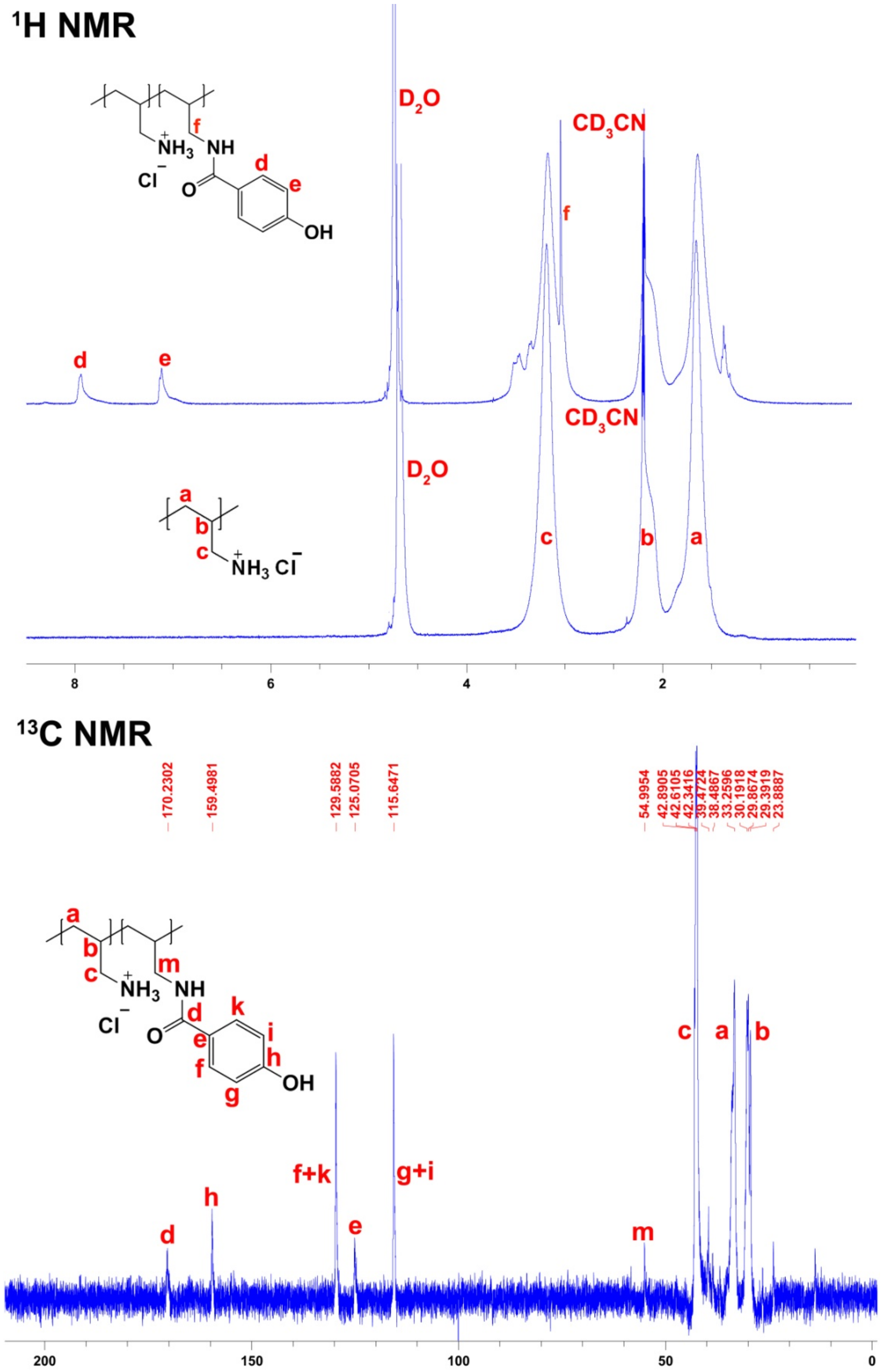

Figure S4. ^{1}H NMR of PAH-*p*HBA polymer, related to Figure 1.

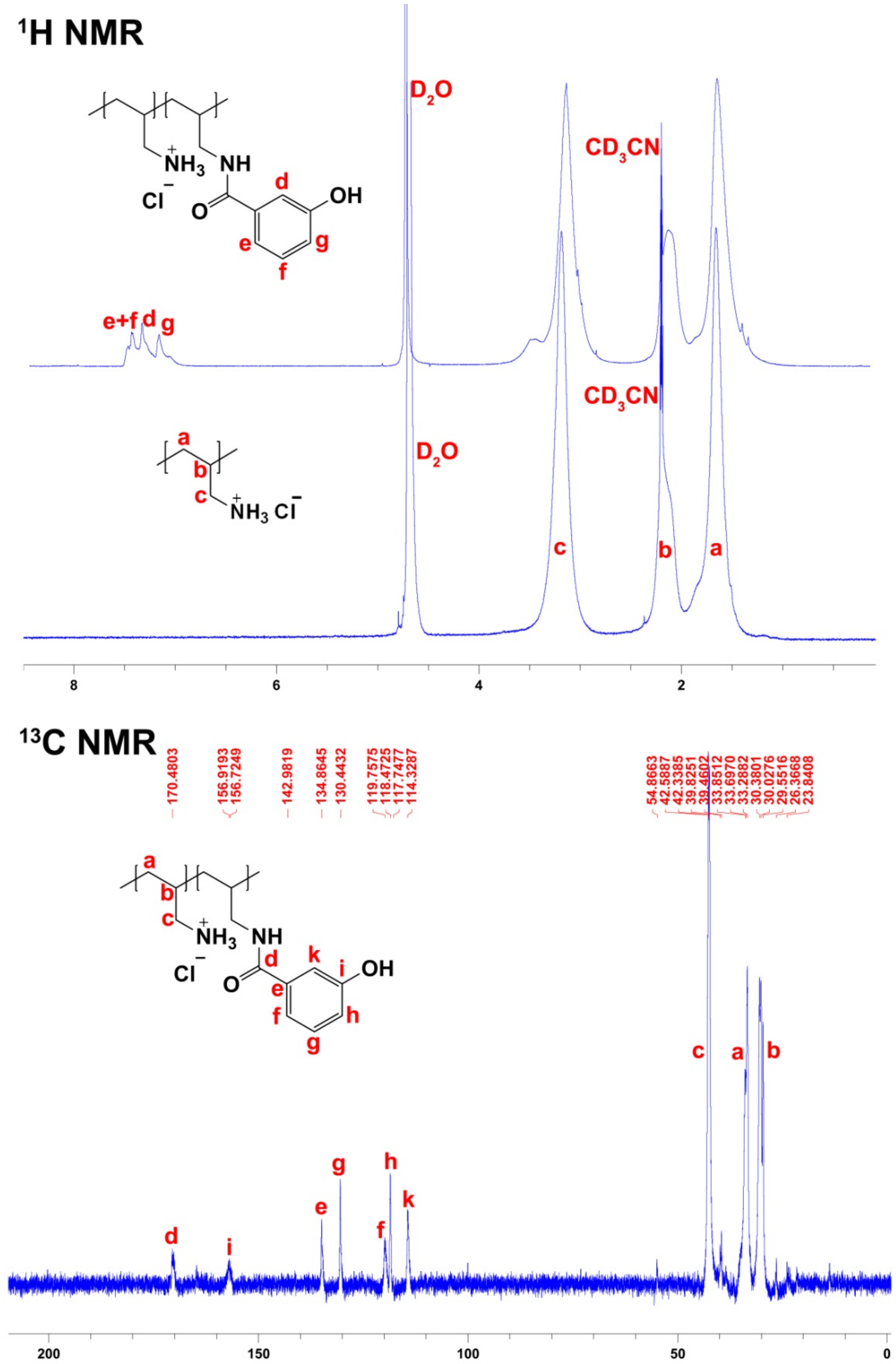

Figure S5. **^{1}H NMR of PAH-*m*HBA polymer, related to Figure 1.**

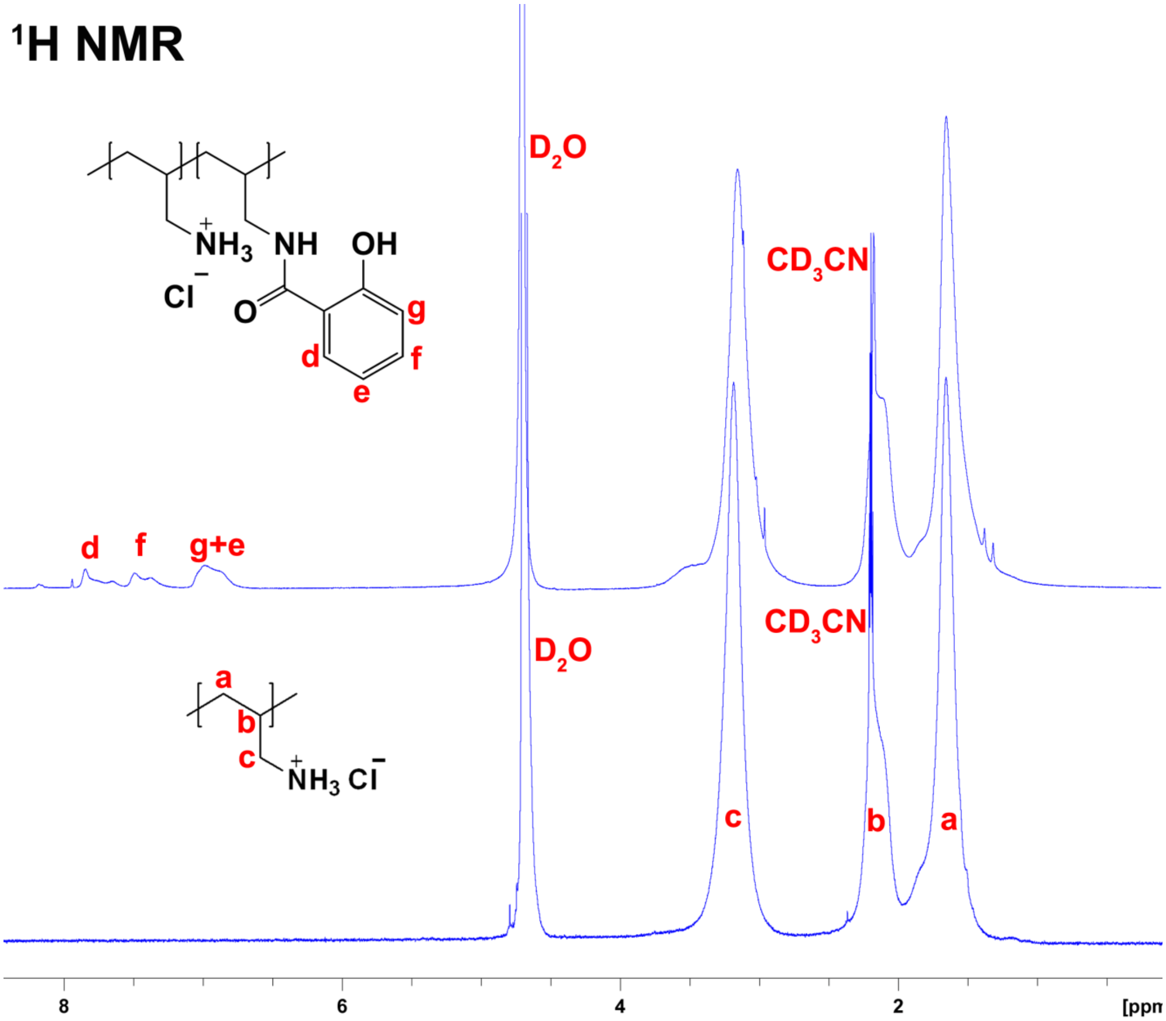

Figure S6. ^{1}H NMR of PAH-*o*HBA polymer, related to Figure 1.

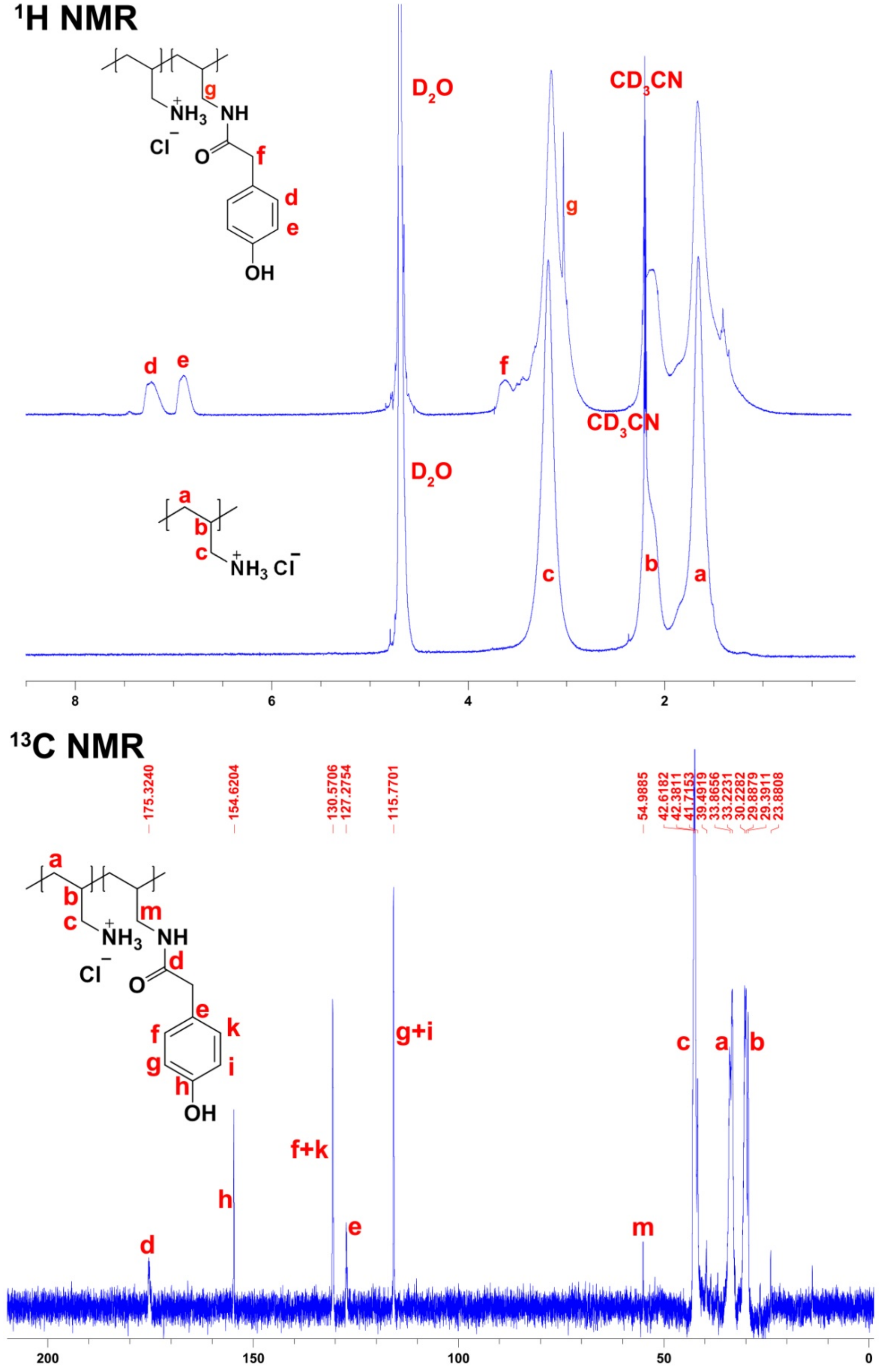

Figure S7. ^{1}H NMR of PAH-*p*HPA polymer, related to Figure 1.

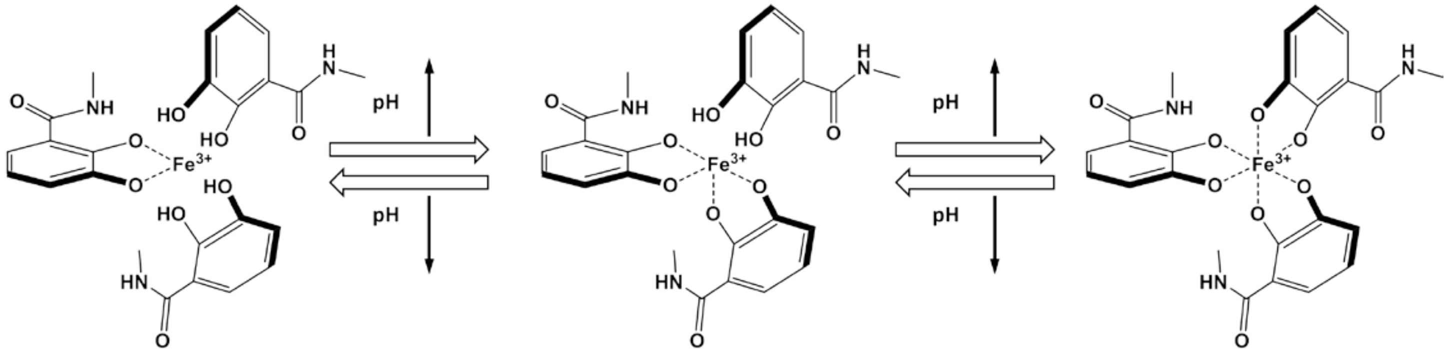

Figure S8. The pH-dependent stoichiometry of Ferric catechol complexes, related to Figure 1.

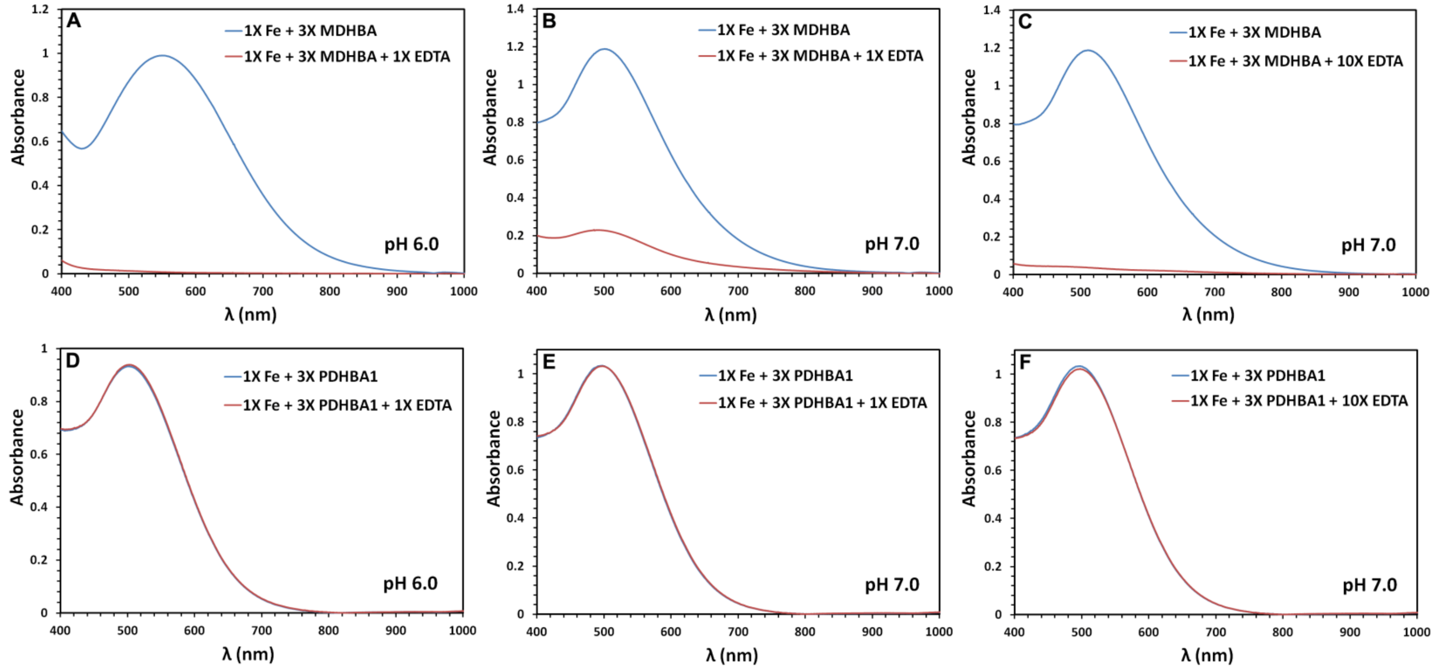

Figure S9. Comparison of the iron affinities of MDHBA and PAH-DHBA1 with the competition of EDTA, related to Figure 1. **A**, **B**, and **C**, Visible spectra change of ferric MDHBA system in the presence of EDTA at different pH. (A) molar ratio of Fe: MDHBA: EDTA = 1: 3: 1 at pH 6.0. (B) molar ratio of Fe: MDHBA: EDTA = 1: 3: 1 at pH 7.0. (C) molar ratio of Fe: MDHBA: EDTA = 1: 3: 10 at pH 7.0. D, E, and F, Visible spectra change of ferric PDHBA1 system in the presence of EDTA at different pH. (D) molar ratio of Fe: PDHBA1: EDTA = 1: 3: 1 at pH 6.0. (E) molar ratio of Fe: PDHBA1: EDTA = 1: 3: 1 at pH 7.0. (F) molar ratio of Fe: PDHBA1: EDTA = 1: 3: 10 at pH 7.0.

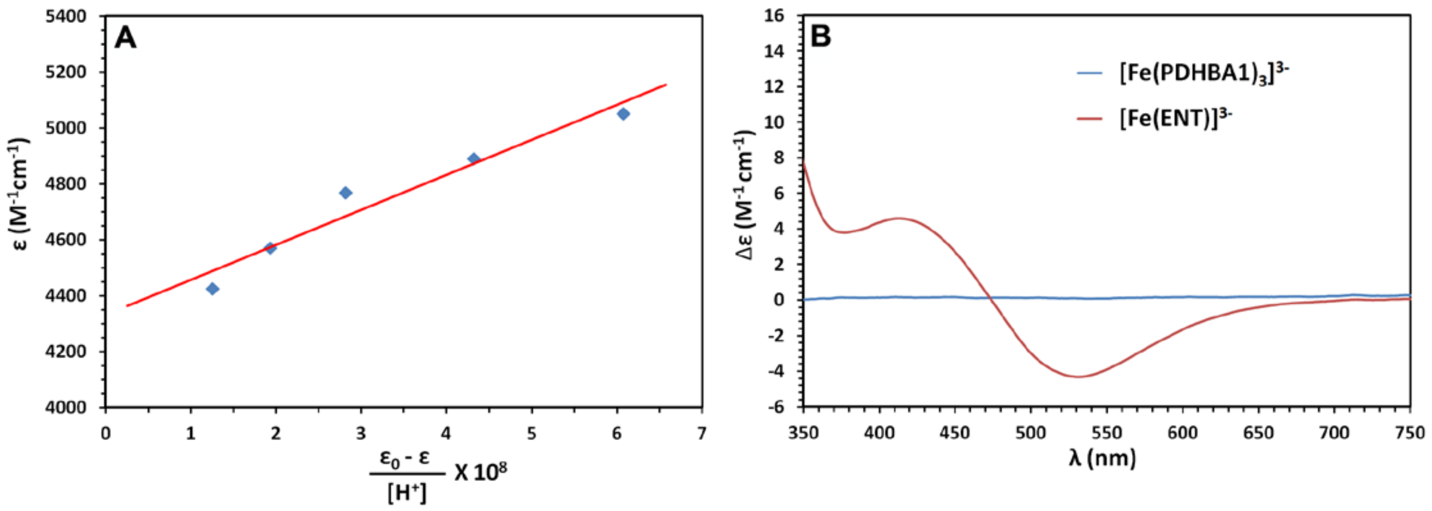

Figure S10. Characterization of polymer iron complexes, related to Figure 1. A. Schwarzenbach plot of (ε_{ML} - ε)/$[H^+]^n$ *vs*. ε for ferric PAH-DHBA1 using n = 1 (A), where ε_{ML} is the molar extinction coefficient of $[Fe(PDHBA1)_3]^{3-}$ at 495 nm and ε is the apparent extinction coefficient at any pH (ε = $absorbance_{495}/([Fe]_{total} \cdot 1$ cm)). The data represent the pH range 5.01-6.02. **B**. Circular dichroism spectra of $[Fe(PDHBA1)_3]^{3-}$ and $[Fe(ENT)]^{3-}$ complexes. Condition: 0.05 mM Fe^{3+}, 0.15 mM PDHBA1 in 1 M KCl solution at pH 7.0 for ferric PDHBA1 system, and 0.05 mM Fe^{3+}, 0.05 mM ENT in 1 M KCl solution at pH 7.0 for ferric ENT system.

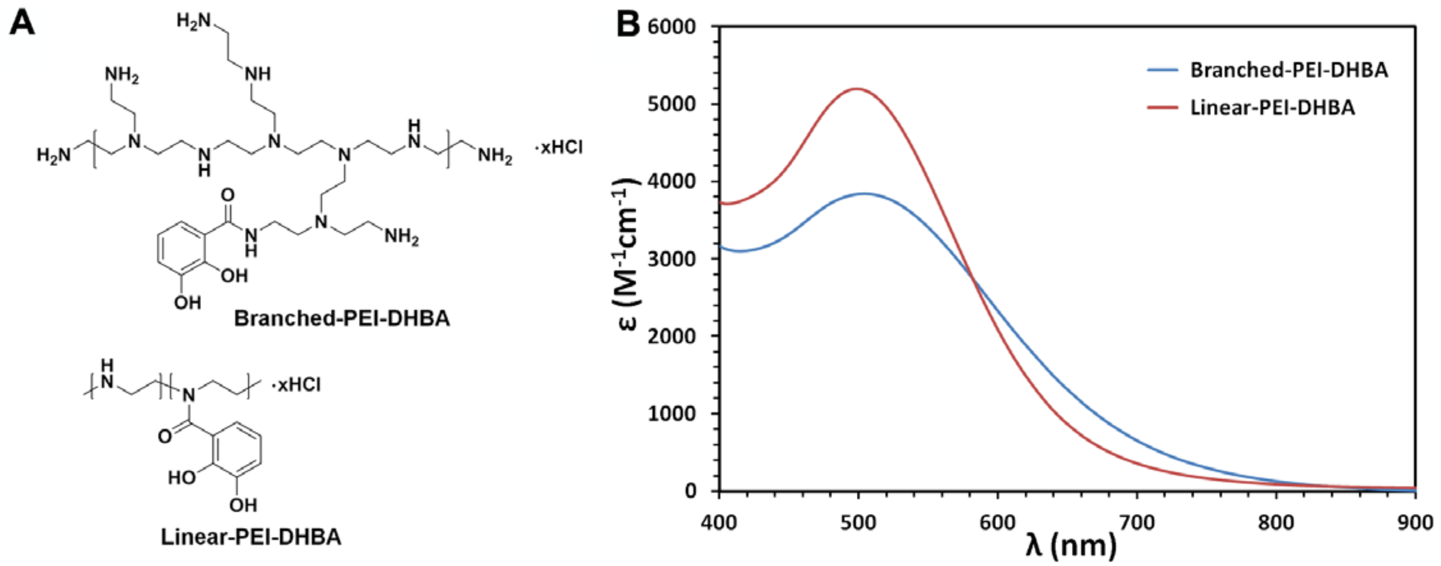

Figure S11. Comparison of ferric Branched-PEI-DHBA and Linear-PEI-DHBA systems, related to Figure 2. **A**. Chemical structures of Branched-PEI-DHBA and Linear-PEI-DHBA polymers. **B**. Visible absorption spectra of ferric Branched-PEI-DHBA and Linear-PEI-DHBA systems at pH 7.0. Condition: 0.05 mM Fe^{3+}, 0.15 mM PDHBA ligands in 1 M KCl solution at pH 7.0.

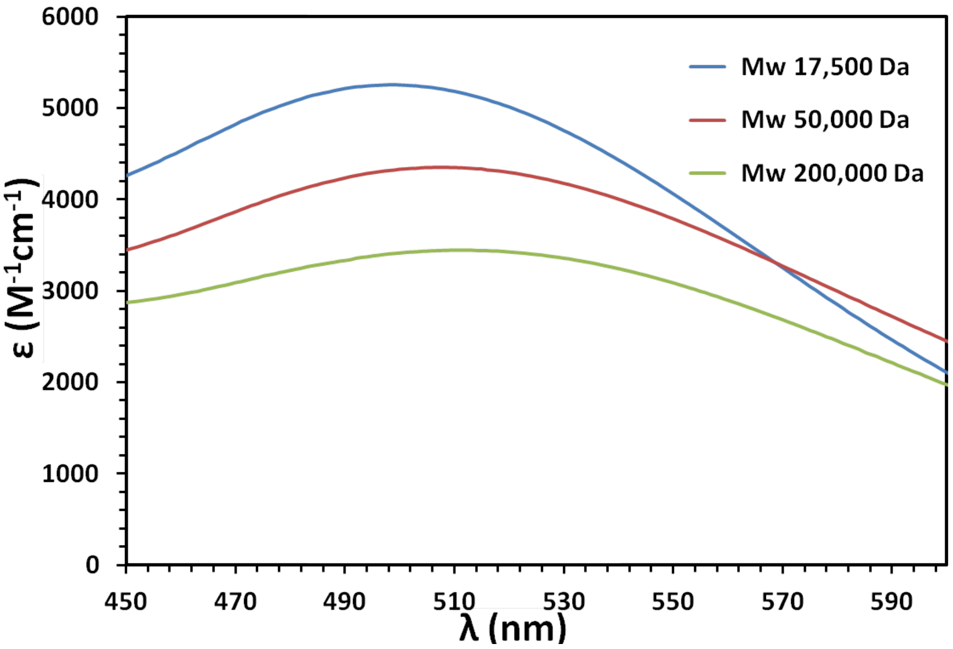

Figure S12. Visible absorption spectra of ferric PAH-DHBA systems with the same DHBA contents (~5%) but different PAH molecular weights, related to Figure 2. Condition: 0.05 mM Fe^{3+}, 0.15 mM PDHBA ligands in 1 M KCl solution at pH 7.0.

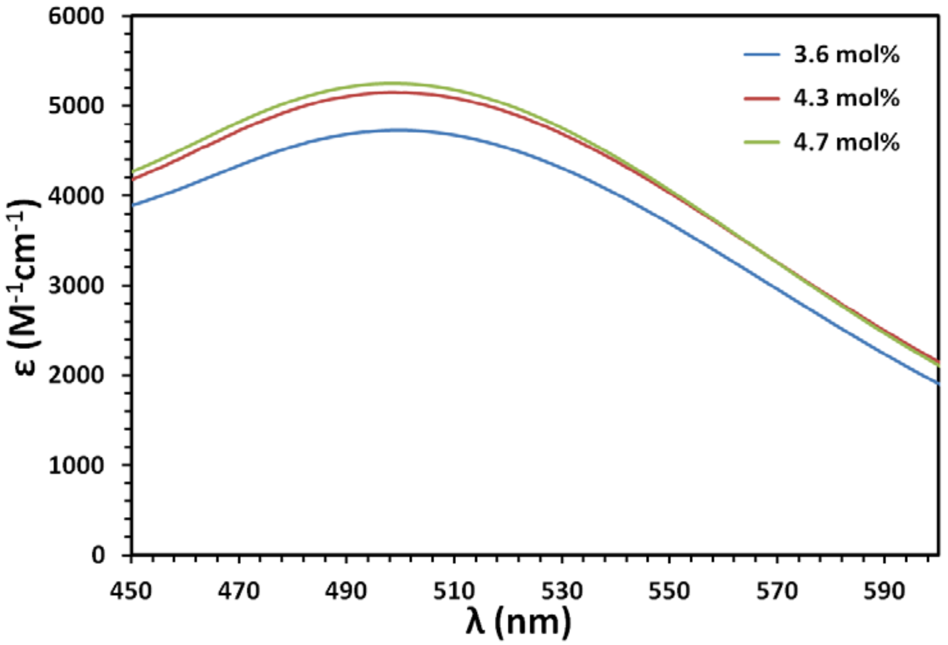

Figure S13. Visible absorption spectra of ferric PAH-DHBA systems with different DHBA contents, related to Figure 2. Condition: 0.05 mM Fe^{3+}, 0.15 mM PDHBA ligands in 1 M KCl solution at pH 7.0.

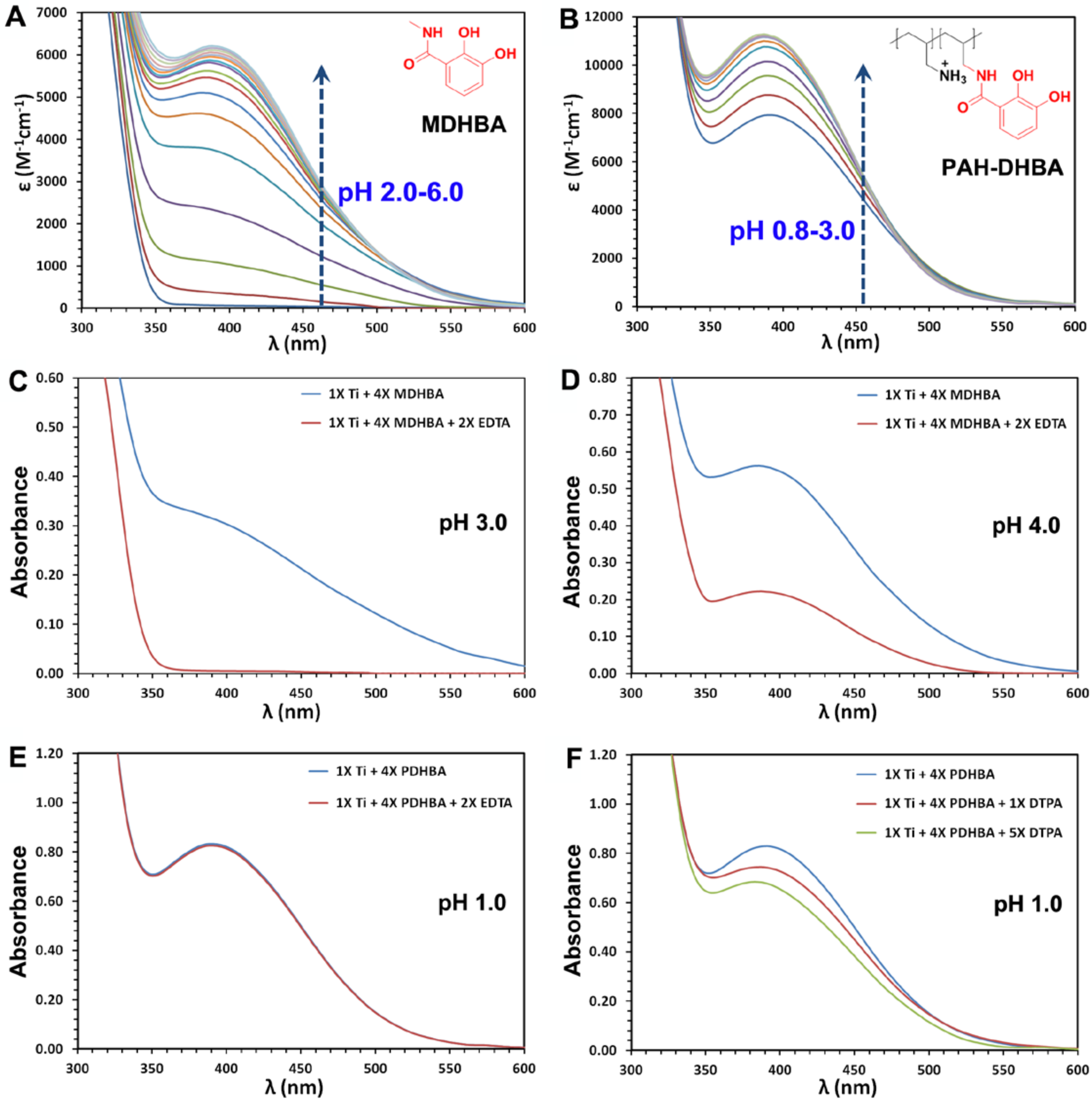

Figure S14. Conformational stability effect was observed in Titanium(IV) PAH-DHBA system, related to Figure 3. Visible spectra of Ti(IV)-MDHBA (A) and Ti(IV)-PDHBA (B) systems as a function of pH. pH ranges: 2.0-6.0 for Ti(IV)-MDHBA system; 0.8-3.0 for Ti(IV)-PDHBA system. Visible spectra change of Ti(IV)-MDHBA system in the presence of EDTA at pH 3.0 (C) and 4.0 (D). Visible spectra change of Ti(IV)-PDHBA system in the presence of EDTA (E) and DTPA (F) at pH 1.0. Condition: 0.1 mM Ti^{4+}, 0.4 mM MDHBA or PDHBA ligands in 1 M KCl solution.

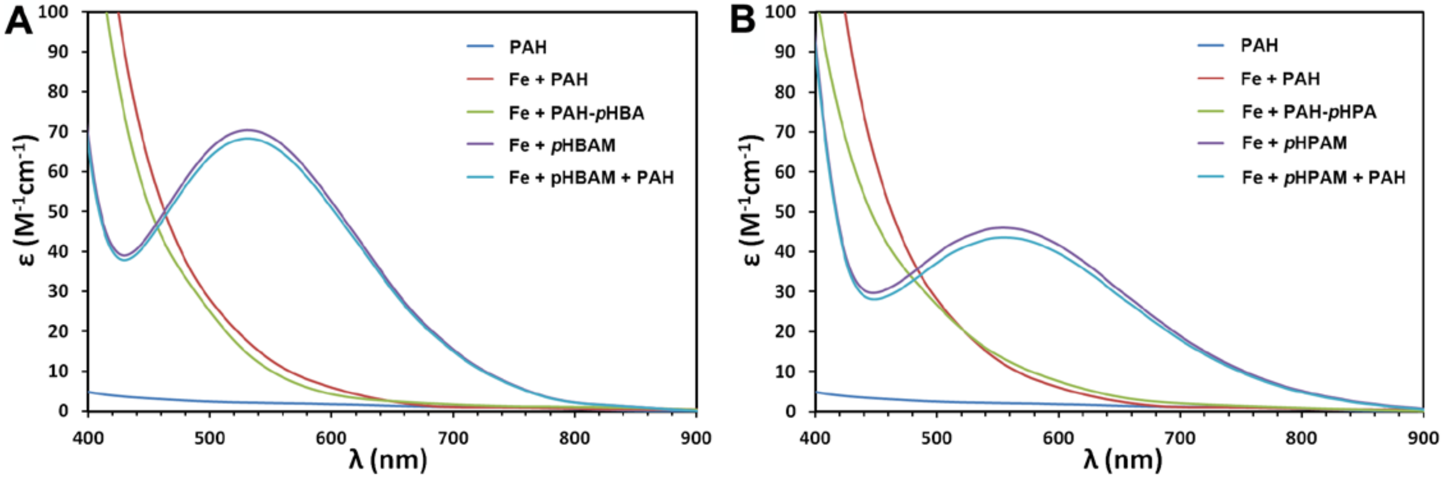

Figure S15. Visible absorption spectra of ferric phenol systems in the presence of PAH, related to Figure 4. A. Visible spectra of ferric *p*HBAM system in the presence of PAH. **B**. Visible spectra of ferric *p*HPAM system in the presence of PAH. Condition: 1 mM Fe^{3+}, 4 mM ligands in 0.1 M KCl solution at pH 2.6. The mass concentration of PAH is the same as the one of PAH-*p*HBA or PAH-*p*HPA. With the presence of PAH, the visible spectra of ferric *p*HBAM and *p*HPAM systems showed no remarkable change, which indicated that the loss of ferric chelation capability did not result from the chelation competition of PAH component.

Transparent methods

Materials

1-Hydroxybenzotriazole (HOBt) was purchased from Chem-Impex International, Inc. Poly(allylamine hydrochloride) (PAH) with average molecular weight 17,500 kDa, N-(3-Dimethylaminopropyl)-N'-ethylcarbodiimide hydrochloride (EDC), 2,3-Dihydroxybenzoic acid (DHBA), 3,4-Dihydroxyhydrocinnamic acid (DHCA), Protocatechuic acid (PCCA), Enterobactin (ENT), 4-Hydroxybenzoic acid (HBA), 4-Hydroxyphenylacetic acid (HPA), and other chemicals were obtained from Sigma-Aldrich. All the chemicals were used as received.

Preparation of Polyallylamine free base

PAH (9.35 g) and NaOH (4.2 g) were dissolved in 60 mL DI water, and then purified through dialysis (MWCO 6,000-8,000 Da). The polyallylamine (free base) was obtained by lyophilization.

Preparation of DHBA conjugated PAH (PAH-DHBA)

Three PAH-DHBA polymer chelators were prepared with different DHBA contents. HOBt-activated DHBA was synthesized before preparing the polymers. Typically, 1 equiv of DHBA, 2 equiv of HOBt, and 1.5 equiv of EDC were dissolved in DMF (DHBA concentration ~25 wt%). The mixture was stirred at room temperature for 8 h and used for the next reaction step without any purification. The detailed amounts of reagents were shown in Table S1.

Table S1. Preparation and characterization of PAH-DHBA polymer chelators

	Polyallylamine amount	DHBA amount	DHBA feed ratio	DHBA found ratio
**PAH-DHBA1**	570 mg	115.5 mg	7.5%	4.7%[a], 4.9%[b]
**PAH-DHBA2**	570 mg	154.0 mg	10.0%	6.5%[a], 6.9%[b]
**PAH-DHBA3**	570 mg	231.0 mg	15.0%	10.0%[a], 10.8%[b]

a, determined by H^1 NMR; b, determined by elemental analysis.

Polyallylamine free base 570 mg (10 mmol amine groups) was dissolved in 120 mL H_2O/DMF (70 mL/50 mL) mixture and purged with nitrogen for 30 min. The as-prepared HOBt-activated DHBA solution was dropwise transferred to the polyallylamine solution under the protection of nitrogen. The mixture was sealed and stirred at room temperature for 24 h. Then 10 mL hydrochloric acid (6 M) was added to the above solution to quench the reaction. PAH-DHBA was purified by dialysis (MWCO 3,500 Da) under the protection of nitrogen and then lyophilized. The DHBA contents were characterized by H^1 NMR and elemental analysis.

Preparation of DHCA conjugated PAH (PAH-DHCA)

HOBt-activated DHCA was synthesized before preparing the polymers. DHCA (124.5 mg), HOBt (203.0 mg), and EDC (220.0 mg) were dissolved in 5 mL DMF. The mixture was stirred at room temperature for 8 h and used for the next reaction step without any purification.

Polyallylamine free base 0.57 g (10 mmol amine groups) was dissolved in 120 mL H_2O/DMF (70 mL/50 mL) mixture and purged with nitrogen for 30 min. The as-prepared HOBt-activated DHCA solution was dropwise transferred to the polyallylamine solution under the protection of nitrogen. The mixture was sealed and stirred at room temperature for 24 h. Then 10 mL hydrochloric acid (6 M) was added to the above solution to quench the reaction. PAH-DHCA was purified by dialysis (MWCO 3,500 Da) under the protection of nitrogen and then lyophilized. The DHCA content was 5.9% determined by H^1 NMR and 6.5% by elemental analysis.

Preparation of PCCA conjugated PAH (PAH-PCCA)

HOBt-activated PCCA was synthesized before preparing the polymers. PCCA (123.2 mg), HOBt (216 mg), and EDC (235 mg) were dissolved in 5 mL DMF. The mixture was stirred at room temperature for 8 h and used for the next reaction step without any purification.

Polyallylamine free base 0.57 g (10 mmol amine groups) was dissolved in 120 mL H_2O/DMF (70 mL/50 mL) mixture and purged with nitrogen for 30 min. The as-prepared HOBt-activated PCCA solution was dropwise transferred to the polyallylamine solution under the protection of nitrogen. The mixture was sealed and stirred at room temperature for 24 h. Then 10 mL hydrochloric acid (6 M) was added to the above solution to quench the reaction. PAH-PCCA was purified by dialysis (MWCO 3,500 Da) under the protection of nitrogen and then lyophilized. The PCCA content was 5.2% determined by H^1 NMR and 5.9% by elemental analysis.

Preparation of *p*HBA conjugated PAH (PAH-*p*HBA)

HOBt-activated *p*HBA was synthesized before preparing the polymers. *p*HBA (110.4 mg), HOBt (216 mg), and EDC (235 mg) were dissolved in 5 mL DMF. The mixture was stirred at room temperature for 5 h and used for the next reaction step without any purification.

Polyallylamine free base 0.57 g (10 mmol amine groups) was dissolved in 120 mL H_2O/DMF (70 mL/50 mL) mixture and purged with nitrogen for 30 min. The as-prepared HOBt-activated *p*HBA solution was dropwise transferred to the polyallylamine solution under the protection of nitrogen. The mixture was sealed and stirred at room temperature for 24 h. Then 10 mL hydrochloric acid (6 M) was added to the above solution to quench the reaction. PAH-*p*HBA was purified by dialysis (MWCO 3,500 Da) under the protection of nitrogen and then lyophilized. The *p*HBA content was 5.0% determined by H^1 NMR and 5.8% by elemental analysis.

Preparation of *m*HBA conjugated PAH (PAH-*m*HBA)

The procedure of preparing PAH-*m*HBA was the same as the one of preparing PAH-*p*HBA. The *m*HBA content was 5.2% determined by H^1 NMR and 5.9% by elemental analysis.

Preparation of *o*HBA conjugated PAH (PAH-*o*HBA)

The procedure of preparing PAH-*o*HBA was the same as the one of preparing PAH-*p*HBA. The *o*HBA content was 4.9% determined by H^1 NMR and 5.8% by elemental analysis.

Preparation of *p*HPA conjugated PAH (PAH-*p*HPA)

HOBt-activated *p*HPA was synthesized before preparing the polymers. *p*HPA (121.6 mg), HOBt (216 mg), and EDC (235 mg) were dissolved in 5 mL DMF. The mixture was stirred at room temperature for 5 h and used for the next reaction step without any purification.

Polyallylamine free base 0.57 g (10 mmol amine groups) was dissolved in 120 mL H_2O/DMF (70 mL/50 mL) mixture and purged with nitrogen for 30 min. The as-prepared HOBt-activated *p*HPA solution was dropwise transferred to the polyallylamine solution under the protection of nitrogen. The mixture was sealed and stirred at room temperature for 24 h. Then 10 mL hydrochloric acid (6 M) was added to the above solution to quench the reaction. PAH-*p*HPA was purified by dialysis (MWCO 3,500 Da) under the protection of nitrogen and then lyophilized. The *p*HPA content was 6.2% determined by H^1 NMR and 6.7% by elemental analysis.

Preparation of cross-linked PAH-DHBA hydrogel

Briefly, a 13% w/w PAH-DHBA1 solution containing a predetermined amount of N,N-methylene bisacrylamide (0.05%, 0.1%, 0.5%, or 1%, molar ratio of cross-linker to total amines of PAH-DHBA1) was prepared in H_2O/DMF (50/50 v/v) mixture. Triethylamine (TEA) was then added to the solution and mixed thoroughly, and the solution was incubated at room temperature with the protection of nitrogen for 48 h. The resultant cross-linked polymers were then washed with 0.5 M hydrochloric acid and subsequently washed with deionized water under the protection of nitrogen and lyophilized.

Spectrophotometric Titrations

For the spectrophotometric titrations of PAH-DHBA, PAH-DHCA, and PAH-PCCA or their corresponding small molecular counterparts (MDHBA, MDHCA, and MPCCA), a stock solution of 1 mM $FeCl_3$ and a stock solution of polymer chelator containing 1 mM PDHBA (or PDHCA, PPCCA) groups were prepared in 1 M KCl solution. Then 0.5 mL of $FeCl_3$ stock solution and 1.5 mL of polymer chelator stock solution were transferred to 8 mL of 1 M KCl solution to obtain a working solution containing 0.05 mM of $FeCl_3$ and 0.15 mM of PDHBA (or PDHCA, PPCCA) groups for spectrophotometric measurements. In the spectrophotometric titrations, the pH was adjusted by adding hydrochloric acid or sodium hydroxide, and after the pH stabilized (less than 5 min), an aliquot was removed for measuring the UV-vis spectrum.

For the spectrophotometric titrations of ENT, a stock solution of 1 mM $FeCl_3$ and a stock solution of 0.2 mM ENT were prepared in 1 M KCl solution. Then 0.2 mL of $FeCl_3$ stock solution and 1 mL of ENT stock solution were transferred to 8.8 mL of 1 M KCl solution to obtain a working solution containing 0.02 mM of $FeCl_3$ and 0.02 mM of ENT for spectrophotometric measurements. The following procedures are the same as described above.

For the spectrophotometric titrations of PAH-*p*HBA, PAH-*m*HBA, and PAH-*o*HBA, a stock solution of 1 mM $FeCl_3$ and a stock solution of polymer chelator containing 1 mM P*p*HBA (or P*m*HBA, P*o*HBA) groups were prepared in 0.1 M KCl solution. Then 0.5 mL of $FeCl_3$ stock solution and 2 mL of polymer chelator stock solution were transferred to 7.5 mL of 0.1 M KCl solution to obtain a working solution containing 0.05 mM of $FeCl_3$ and 0.2 mM of P*p*HBA (or P*m*HBA, P*o*HBA) groups for spectrophotometric measurements. The following procedures are the same as described above.

For the titanium(IV) PAH-DHBA system, all the procedures are the same as described above, except that the working solution prepared contained 0.1 mM of $TiCl_4$ and 0.4 mM of PDHBA.

Schwarzenbach equation

Any simple protonation reaction of only two species with unknown stoichiometry can be described by the following chemical equation.

$$ML + nH^+ \rightleftharpoons MH_nL$$

$$K = [MH_nL]/[ML][H^+]^n \qquad (1)$$

$$[M]_{total} = [MH_nL] + [ML] \qquad (2)$$

$$Abs = \varepsilon_{ML}\,[ML] + \varepsilon_{MHnL}\,[MH_nL] \qquad (3)$$

$$\varepsilon = Abs/([M]_{total} \cdot 1\ cm) \qquad (4)$$

$$\varepsilon = \varepsilon_{MHnL} + (\varepsilon_{ML} - \varepsilon)/K[H^+]^n \qquad (5)$$

These equations can be rearranged to give equation 5. As long as only two species are present, which can be indicated by an isosbestic point, a plot of ε *vs*. $1/[H^+]^n$ should be linear by selecting an appropriate value of n, which is the stoichiometry of the hydrogen ion termed in the above chemical equation.

Determination of the iron stability constants

The iron stability constants for polymer chelators and ENT were measured by a ligand competition assay using EDTA as a competitor. For measuring stability constants of PAH-DHBA, PAH-PCCA, or PAH-DHCA polymers, a solution containing 0.1 mM of $FeCl_3$, 0.3 mM of corresponding ligands (PDHBA, PPCCA, or PDHCA) on the polymers, 0.8 mM EDTA, and 1 M KCl was prepared. The pH values of the solutions were adjusted to about 5.0, 6.0, and 7.0 for PAH-DHBA, PAH-PCCA, and PAH-DHCA systems, respectively. Then, the solution was equilibrated in the dark at room temperature for 3 h (shortened to 1 h for PAH-DHCA system due to the easy oxidation of PDHCA groups). Then the concentration of the polymer-iron complex after equilibrium was determined by UV-Vis spectrometry. For the measurement of the stability constants of the small molecular counterparts, a solution containing 0.1 mM of $FeCl_3$, 0.4 mM of ligands (MDHBA, MPCCA, or MDHCA), 0.1 mM EDTA, and 1 M KCl was prepared. The pH values of the solutions were adjusted to about 7.0, 8.0, and 8.5 for PAH-DHBA, PAH-PCCA, and PAH-DHCA systems, respectively. Then, the solution was equilibrated in the dark at room temperature for 3 h (shortened to 20 min for MDHCA system due to the easy oxidation of MDHCA).

Take PAH-DHBA system as an example, in the equilibrium situation, the system could be represented in by the following reaction:

$$[Fe(EDTA)]^- + 3\ PDHBA^{2-} \rightleftharpoons [Fe(PDHBA)_3]^{3-} + EDTA^{4-}$$

$$Kq = \frac{[Fe(PDHBA)_3{}^{3-}][EDTA^{4-}]}{[Fe(EDTA)^-][PDHBA^{2-}]^3} = \frac{[Fe(PDHBA)_3{}^{3-}]}{[Fe^{3+}][PDHBA^{2-}]^3} \times \frac{[Fe^{3+}][EDTA^{4-}]}{[Fe(EDTA)^-]} \qquad (1)$$

The iron stability constant of PDHBA (K) could be defined as follows:

$$K = \frac{[Fe(PDHBA)_3{}^{3-}]}{[Fe^{3+}][PDHBA^{2-}]^3} \qquad (2)$$

The iron stability constant of EDTA (K’) could be defined as:

$$K' = \frac{[Fe(EDTA)^-]}{[Fe^{3+}][EDTA^{4-}]} \qquad (3)$$

Based on equation (1), (2), and (3) $\quad \mathrm{LogK} = \mathrm{Log}(Kq \times K') = \mathrm{LogKq} + \mathrm{LogK'}$

The value of K’ for EDTA was known (LogK’ 25.1), and Kq could be calculated based on equation (1). Two assumptions were made for the calculation. Firstly, as the total EDTA concentration was higher than total Fe^{3+} concentration, we assumed that there was no free iron in solution due to the strong iron affinity of EDTA. Secondly, we assumed that only the most stable $[Fe(PDHBA)_3]^{3-}$ complexes existed in the equilibrium at sufficient EDTA concentration, and the less stable $[FeH(PDHBA)_3]^{2-}$ complexes were all disassociated by the competition of EDTA.

Based on the second assumption, the concentration of $[Fe(PDHBA)_3]^{3-}$ could be directly calculated by the Beer-Lambert law as: $Abs_{493}/(l\cdot\varepsilon)$. Abs_{493} is the absorbance at 493 nm, l is the light path (1 cm), and ε is the molar extinction coefficient of $[Fe(PDHBA)_3]^{3-}$ complex (5650 $M^{-1}cm^{-1}$). Based on the first assumption, there was no free Fe^{3+} in solution. The concentration of $[Fe(EDTA)]^-$ was calculated as the total iron concentration minus the determined concentration of $[Fe(PDHBA)_3]^{3-}$. The concentration of EDTA (the sum concentrations of consecutive protonated species of EDTA, including H_6EDTA^{2+}, H_5EDTA^+, H_4EDTA, H_3EDTA^-, H_2EDTA^{2-}, $HEDTA^{3-}$, and $EDTA^{4-}$) in the solution after competition was calculated as the total EDTA concentration subtracted by the concentration of $[Fe(EDTA)]^-$. The concentration of PDHBA groups (the sum concentrations of consecutive protonated species of PDHBA, including H_2PDHBA, $HPDHBA^-$, and $PDHBA^{2-}$) could be calculated as: total concentration of PDHBA groups minus three times of the $[Fe(PDHBA)_3]^{3-}$ concentration. In the calculation, the pKa values of EDTA and PDHBA were also required to calculate the concentrations of $EDTA^{4-}$ and $PDHBA^{2-}$ ions.

The fraction of $EDTA^{4-}$ is

$$x_{EDTA^{4-}} = \frac{K_1K_2K_3K_4K_5K_6}{[H^+]^6 + [H^+]^5K_1 + [H^+]^4K_1K_2 + [H^+]^3K_1K_2K_3 + [H^+]^2K_1K_2K_3K_4 + [H^+]K_1K_2K_3K_4K_5 + K_1K_2K_3K_4K_5K_6}$$

For EDTA, pK1 = 0.0, pK2 = 1.5, pK3 = 2.0, pK4 = 2.66, pK5 = 6.16, pK6 = 10.24.

The fraction of $PDHBA^{2-}$ is

$$x_{PDHBA^{2-}} = \frac{K_1K_2}{[H^+]^2 + [H^+]K_1 + K_1K_2}$$

For the pK1 and pK2 of PDHBA group, we assumed that they were almost the same as the ones of its small molecular counterpart MDHBA, since they both share the same functional structure.

All the assumptions and calculation methods employed for the determination of the iron stability constant of PAH-DHBA could be directly used to determine the stability constants of PAH-PCCA and PAH-DHCA polymer chelators.

The pK1 and pK2 values for MDHBA, MPCCA, and MDHCA determined by potentiometric titration are listed below:

MDHBA: pK1 7.5, pK2 11.3

MPCCA: pK1 8.1, pK2 12.7

MDHCA: pK1 8.8, pK2 13.2

The molar extinction coefficients of $[Fe(PDHBA)_3]^{3-}$, $[Fe(PPCCA)_3]^{3-}$, and $[Fe(PDHCA)_3]^{3-}$ complexes determined by visible spectrometry are 5650, 5940, and 5200 $M^{-1}cm^{-1}$, respectively.

For measuring iron stability constants ENT, a solution containing 0.02 mM of $FeCl_3$, 0.02 mM of ENT, 0.16 mM EDTA, and 1 M KCl was prepared. After the pH adjusted to about 5.0, the solution was equilibrated in the dark at room temperature for 3 h. Then the concentration of the $[FeENT]^{3-}$ after equilibrium was determined by UV-Vis spectrometry.

In the equilibrium situation, the system could be represented in by the following reaction:

$$Fe(EDTA)^- + ENT^{6-} \rightleftharpoons [Fe(ENT)]^{3-} + EDTA^{4-}$$

$$Kq = \frac{[Fe(ENT)^{3-}][EDTA^{4-}]}{[Fe(EDTA)^-][ENT^{6-}]} = \frac{[Fe(ENT)^{3-}]}{[Fe^{3+}][ENT^{6-}]} \times \frac{[Fe^{3+}][EDTA^{4-}]}{[Fe(EDTA)^-]} \qquad (4)$$

The iron stability constant of DHBA (K) could be defined as follows:

$$K = \frac{[Fe(ENT)^{3-}]}{[Fe^{3+}][ENT^{6-}]} \qquad (5)$$

The iron stability constant of EDTA (K') could be defined as:

$$K' = \frac{[Fe(EDTA)^-]}{[Fe^{3+}][EDTA^{4-}]} \qquad (6)$$

Based on equation (4), (5), and (6):

$$\mathrm{LogK} = \mathrm{Log}(\mathrm{Kq} \times \mathrm{K'}) = \mathrm{LogKq} + \mathrm{LogK'}$$

The value of K' was known, and Kq could be easily calculated based on equation (4). The calculation method was similar with the one mentioned above. The pK1, pK2, pK3, pK4, pK5, and pK6 values are 7.6, 8.4, 9.2, 11.3, 12.1, and 12.9, respectively[1]. The molar extinction coefficient of $[Fe(ENT)_3]^{3-}$ complex is 5700 $M^{-1}cm^{-1}$ determined by visible spectrometry.

Isothermal titration calorimetry studies

ITC studies were carried out on a MicroCal VP-ITC (Malvern UK). All experiments were carried out at 25 °C in 100 mM HEPES buffer, pH 7.2 ± 0.2. Titrations were carried out by injecting 20 consecutive 10 μL aliquots of 1 mM Fe-EDTA (Fe^{3+} 1 mM, EDTA 1 mM) into the sample cell containing 1.4119 mL of 0.2 mM of PDHBA ligands on polymer chelators or 0.0667 mM of ENT. The time between first 4 and the following injections was 12 and 30 min, respectively.
